# Matrix association region/scaffold attachment region (MAR/SAR) sequence: its vital role in mediating chromosome breakages in nasopharyngeal epithelial cells via oxidative stress-induced apoptosis

**DOI:** 10.1186/s12867-018-0116-5

**Published:** 2018-12-04

**Authors:** Sang-Nee Tan, Sai-Peng Sim, Alan S. B. Khoo

**Affiliations:** 1 Department of Paraclinical Sciences, Faculty of Medicine and Health Sciences, Universiti Malaysia Sarawak, Kota Samarahan, Sarawak Malaysia; 2 Molecular Pathology Unit, Cancer Research Centre, Institute for Medical Research, Kuala Lumpur, Malaysia

**Keywords:** NPC, Oxidative stress, H_2_O_2_, Apoptosis, MAR/SAR, AF9 gene

## Abstract

**Background:**

Oxidative stress is known to be involved in most of the aetiological factors of nasopharyngeal carcinoma (NPC). Cells that are under oxidative stress may undergo apoptosis. We have previously demonstrated that oxidative stress-induced apoptosis could be a potential mechanism mediating chromosome breakages in nasopharyngeal epithelial cells. Additionally, caspase-activated DNase (CAD) may be the vital player in mediating the chromosomal breakages during oxidative stress-induced apoptosis. Chromosomal breakage occurs during apoptosis and chromosome rearrangement. Chromosomal breakages tend to cluster in certain regions, such as matrix association region/scaffold attachment region (MAR/SAR). We hypothesised that oxidative stress-induced apoptosis may result in chromosome breaks preferentially at the MAR/SAR sites. The *AF9* gene at 9p22 was targeted in this study because 9p22 is a deletion site commonly found in NPC.

**Results:**

By using MAR/SAR recognition signature (MRS), potential MAR/SAR sites were predicted in the *AF9* gene. The predicted MAR/SAR sites precisely match to the experimentally determined MAR/SARs. Hydrogen peroxide (H_2_O_2_) was used to induce apoptosis in normal nasopharyngeal epithelial cells (NP69) and NPC cells (HK1). Nested inverse polymerase chain reaction was employed to identify the *AF9* gene cleavages. In the SAR region, the gene cleavage frequency of H_2_O_2_-treated cells was significantly higher than that of the non-treated cells. A few chromosomal breakages were detected within the *AF9* region which was previously found to be involved in the mixed lineage leukaemia (*MLL*)-*AF9* translocation in an acute lymphoblastic leukaemia patient. As for the non-SAR region, no significant difference in the gene cleavage frequency was found between the untreated control and H_2_O_2_-treated cells. Furthermore, H_2_O_2_-induced cleavages within the SAR region were reduced by caspase-3 inhibitor, which indirectly inhibits CAD.

**Conclusions:**

These results reaffirm our previous findings that oxidative stress-induced apoptosis could be one of the potential mechanisms underlying chromosome breakages in nasopharyngeal epithelial cells. MAR/SAR may play a vital role in defining the location of chromosomal breakages mediated by oxidative stress-induced apoptosis, where CAD is the major nuclease.

**Electronic supplementary material:**

The online version of this article (10.1186/s12867-018-0116-5) contains supplementary material, which is available to authorized users.

## Background

Nasopharyngeal carcinoma (NPC) is a type of malignant solid tumour which has been associated with multiple factors. One of the strong risk factors of NPC is Epstein–Barr virus (EBV) infection [1, 2]. A previous study has reported increased levels of immunoglobulin G (IgG) and immunoglobulin A (IgA) antibodies to EBV viral capsid antigen (VCA) and early antigen (EA) in NPC patients [1]. Besides, a case–control study of NPC among Malaysian Chinese has reported that salted fish consumption during childhood was a significant risk factor for developing NPC [3]. Mutagenic activity has been detected in the urine collected from experimental rats that were regularly fed with salted fish [4]. In addition, Chinese salted fish was found to cause nasal cavity tumours in rats [5, 6]. These findings suggested the presence of carcinogenic substances in salted fish. Most studies pointed toward nitrosamines and nitrosamine precursors, which have been recognised as animal carcinogens [7, 8]. In addition, long-term exposures to intense industrial heat, formaldehyde, cigarette smoke and wood dust have also been found to be significantly associated with NPC [3, 9–12]. Recently, chronic inflammation of sinonasal tract has been increasingly recognised as a risk factor for NPC [13, 14].

It is important to note that, all of these aetiological factors provoke the production of reactive oxygen species (ROS) [15–20]. Additionally, ROS was found to be involved in EBV reactivation in NPC cells after treatment with *N*-methyl-*N*′-nitro-*N*-nitroguanidine (MNNG) [21]. The ROS-mediated EBV reactivation was inhibited by apigenin which has been suggested to be a potent ROS scavenger [22]. Increased ROS may cause DNA double-strand breaks and error-prone repair. This may in turn lead to genomic instability [23]. The cancer cells and inflammatory cells in stroma of NPC patients have been found to contain oxidative and nitrative DNA lesions [24]. Oxidative stress may trigger apoptosis, a process of programmed cell death [25]. We have previously demonstrated that oxidative stress-induced apoptosis resulted in chromosomal breakages in normal nasopharyngeal epithelial and NPC cells. In addition, the apoptotic nuclease, caspase-activated DNase (CAD) may be a major player in mediating these chromosomal breakages [26].

Chromosomal breakage is an early event in both apoptotic DNA fragmentation and chromosome rearrangement. Previous studies revealed that chromosome breaks tend to fall within certain regions which contain specific chromatin structural elements, such as the matrix attachment region/scaffold attachment region (MAR/SAR) [27, 28]. MAR/SAR is the DNA sequence where the DNA loop structure binds to nuclear scaffold/matrix proteins [29]. In the early stage of apoptosis, DNA cleavages take place at the base of the DNA loop [30, 31]. We hypothesised that H_2_O_2_-induced apoptosis may cause chromosomal breakages at MAR/SAR resulting in chromosome rearrangement in nasopharyngeal epithelial cells.

This study focuses on the *AF9* gene which is located at 9p22 because 9p22 is one of the deletion hotspots in NPC [32]. The *AF9* gene is 280,880 bp in length. The nucleotide position of its exons and introns are shown in Additional file 1. Strissel et al. have identified two MAR/SARs within the *AF9* gene. These two MAR/SARs were designated as SAR1 and SAR2 [28].

In the present study, in silico prediction of MAR/SAR sites was performed in the *AF9* gene. It was found that in the region that contains MAR/SAR (SAR region), the gene cleavage frequency of H_2_O_2_-treated cells was higher than that of the untreated control. On the contrary, in the region that does not contain MAR/SAR (non-SAR region), there was no significant difference in gene cleavage frequency between untreated and H_2_O_2_-treated cells. These observations are true for both normal nasopharyngeal epithelial and NPC cells. Moreover, the oxidative stress-induced chromosome breakages within the SAR region were reduced by caspase-3 inhibitor, which indirectly inhibits CAD. Our results suggested that MAR/SAR may play an important role in defining the location of chromosome breaks mediated by oxidative stress-induced apoptosis, where CAD is the essential nuclease. These chromosomal breakages may in turn lead to chromosome aberrations in nasopharyngeal epithelial cells.

## Methods

### Cell lines and chemicals

NP69 normal nasopharyngeal epithelial cell line and HK1 NPC cell line were kindly provided by Prof. Tsao Sai Wah (The University of Hong Kong, Hong Kong, China) and Prof. Lo Kwok Wai (The Chinese University of Hong Kong, Hong Kong, China). StemPro ACCUTASE Cell Dissociation Reagent, Keratinocyte-SFM medium, RPMI 1640 medium, penicillin, streptomycin, l-glutamine and fetal bovine serum were purchased from GIBCO, Invitrogen, USA. Camptothecin (CPT) was purchased from Santa Cruz Biotechnology, California, USA. Hydrogen peroxide (H_2_O_2_) was bought from MP Biomedicals, USA. Annexin V-Fluorescein isothiocyanate (FITC) Apoptosis Detection Kit I (BD Pharmingen™) and Flow Cytometry Mitochondrial Membrane Potential Detection Kit (BD™MitoScreen) were obtained from Becton–Dickinson Biosciences, USA. Caspase-Glo 3/7 Assay Kit and dNTP mix were purchased from Promega, USA. Caspase-3 inhibitor II (Z-DEVD-FMK) was obtained from Calbiochem, USA. Isoamyl alchohol was procured from Fluka, Switzerland. Sodium dodecyl sulfate (SDS) and phenol were bought from Amresco, USA. Ammonium acetate was from Merck, Germany. Chloroform was obtained from R&M Chemicals, UK. All the restriction enzymes, T4 DNA Ligase and DNA Polymerase I Large (Klenow) Fragment were purchased from New England Biolabs (NEB), USA. QIAquick Gel Extraction Kit and QIAquick Nucleotide Removal Kit were obtained from QIAGEN, Germany. Phusion High-Fidelity DNA Polymerase was obtained from Finnzymes, Finland. PCR primers were bought from First Base Laboratories.

### In silico prediction of MAR/SARs

The whole sequence of the *AF9* gene was retrieved from Ensembl database [EMBL:ENSG00000171843]. The locations of experimentally isolated MAR/SAR, which were found within the *AF9* gene, were determined from the previous reports [27, 28]. Possible MAR/SAR sites were also identified using MAR/SAR recognition signature (MRS) which was suggested to be strongly associated with MAR/SAR [33]. This MAR/SARs prediction was performed by using DNASTAR software (Lasergene, USA). The MRS comprises two nucleotide motifs that are within 200 bp apart. The first nucleotide motif is an 8 bp degenerate sequence, AATAAYAA while the second nucleotide motif is a 16 bp degenerate sequence, AWWRTAANNWWGNNNC, where Y = C or T; W = A or T; R = A or G; N = A, C, G or T. No mismatch is allowed within the 8 bp sequence whereas one mismatch is allowed within the 16 bp sequence. These two degenerate sequences should be within 200 bp apart. Each sequence can be aligned on either the Watson strand or the Crick strand. Either sequence can precede the other sequence. The sequences may even be overlapping. Clusters of more than one motif of either 8 or 16 bp within 200 bp apart are considered as a single MRS. Moreover, clusters of more than one MRS within close proximity are regarded as a single potential MAR/SAR site. The locations of the presently predicted MAR/SARs were compared with the locations of the experimentally extracted MAR/SARs reported in previous studies [27, 28].

In our in silico prediction of MAR/SAR which had been performed in the Abelson murine leukaemia viral oncogene homolog 1 (*ABL*) gene, there was only one MAR/SAR site predicted in the experimentally isolated SAR. The distance between 8 bp sequence element and the 16 bp sequence element was 248 bp (data not shown). Therefore, in this study, the maximal distance between 8 bp sequence element and the 16 bp sequence element was set at 250 bp.

### Cell cultures

NP69 cells were grown in Keratinocyte-SFM medium supplemented with 4–5 ng/ml recombinant Epidermal Growth Factor (rEGF), 40–50 µg/ml Bovine Pituitary Extract (BPE), 100 U/ml penicillin, 100 µg/ml streptomycin and 2% (v/v) heat-inactivated fetal bovine serum. HK1 cells were cultured in RPMI 1640 medium supplemented with 2 mM l-glutamine, 100 U/ml penicillin, 100 µg/ml streptomycin and 10% (v/v) heat-inactivated fetal bovine serum. Cells were incubated at 37 °C with 5% CO_2_.

### Detection of phosphatidylserine (PS) externalisation

NP69 cells (1.5 × 10^5^) were plated in 150 mm culture dishes and allowed to grow for 48 h. The NP69 cells were incubated with 100 µM of H_2_O_2_ for 16 and 24 h. HK1 cells (5.5 × 10^5^) were seeded in 150 mm culture dishes and allowed to grow for 72 h. The HK1 cells were incubated with 50 µM of H_2_O_2_ for 4 and 8 h. NP69 and HK1 cells treated with camptothecin (CPT) were used as positive controls. After exposure, the cells were harvested by using StemPro ACCUTASE Cell Dissociation Reagent. Annexin V-FITC Apoptosis Detection Kit I was used to detect PS externalisation in the harvested cells as previously described [26].

### Detection of mitochondrial membrane potential (MMP) disruption

NP69 and HK1 cells were treated and harvested as described above. Flow Cytometry Mitochondrial Membrane Potential Detection Kit was used to detect the loss of MMP in the harvested cells as previously described [26].

### Nested IPCR detection of oxidative stress-induced chromosome breaks

#### H_2_O_2_ treatment and genomic DNA extraction

NP69 cells (2 × 10^4^) were plated in 60 mm culture dishes and allowed to grow for 48 h. The NP69 cells were incubated with H_2_O_2_ at concentration of 100 µM for 16 h. HK1 cells (8 × 10^4^) were seeded in 60 mm culture dishes and allowed to grow for 72 h. The HK1 cells were incubated with H_2_O_2_ at concentration of 50 µM for 8 h. After treatment with H_2_O_2_, genomic DNA extraction was carried out as previously described [26].

#### Manipulation of genomic DNA and nested IPCR for the AF9 SAR region

The extracted genomic DNA was manipulated in preparation for nested IPCR as previously described [26]. Additional file 2 shows the simplified manipulation steps. Briefly, *Bam*H I digestion, Klenow fill-in, cyclisation and ethanol precipitation were performed. The DNA was then either digested with *Kpn* I or *Nde* I. QIAGEN QIAquick Nucleotide Removal Kit was used to clean up the DNA. Nested IPCR was carried out as previously reported [26].

#### Manipulation of genomic DNA and nested IPCR for the AF9 non-SAR region

The manipulation steps were similar to the SAR region, except that *Hin*d III (RE2 in Additional file 2) and *Xba* I (RE3 in Additional file 2) were used for the *AF9* non-SAR region instead of *Kpn* I and *Nde* I. Cycle condition used in the first round of IPCR was as below: 30 s of 98 °C for 1 cycle (initial denaturation), followed by 30 cycles of 98 °C for 10 s (denaturation), 64 °C for 30 s (annealing), 72 °C for 22 s (extension), followed by 1 cycle of 72 °C for 10 min (final extension). Two microliter of fivefold diluted first round IPCR product was used for second round with similar cycle condition, except that the annealing temperature was 63 °C and the extension time was 15 s. The primers used for the first round of IPCR were 5′-TACCAAACATTTTGAGTCCTACAG-3′ (reverse) and 5′-GGCATTCAGGTGAGTAGTTTATTC-3′ (forward), whereas the primers used in the second round were 5′-AGCAGTAGACTTTTGTAACCTCAC-3′ (reverse) and 5′-AGGGGATGACTTTTCTTCAATC-3′ (forward).

### Inhibition of caspase by Z-DEVD-FMK

HK1 cells (8 × 10^4^) were seeded in 60 mm culture dishes and grown until 60–70% confluency. HK1 cells were either left untreated or pretreated with 50 μM of Z-DEVD-FMK for 1 h. The HK1 cells were then either left untreated or co-treated with 50 μM of H_2_O_2_ for 8 h. After incubation, genomic DNA was extracted as previously described [26]. Following that, IPCR identification of the chromosome breaks within the *AF9* SAR and non-SAR regions was performed as described above.

### Visualisation and DNA sequencing of the IPCR products

The IPCR products were loaded on 1% agarose gel. To analyse the IPCR bands, the gel was stained with ethidium bromide and visualised on an ultraviolet (UV) transilluminator (Vilber Lourmat, USA). QIAGEN QIAquick Gel Extraction Kit was used to clean up the IPCR bands which represent the cleaved fragments derived from the *AF9* SAR region. The purified IPCR bands were sequenced. By blasting the human genome database (Nucleotide BLAST, http://blast.ncbi.nlm.nih.gov/Blast.cgi), the sequencing results were annotated. To identify the position of the chromosome breaks, the sequencing results were aligned with the *AF9* gene sequence accessed from EMBL database [EMBL:ENSG00000171843] by using Seqman DNASTAR software (Lasergene, USA). The breakpoints identified were compared with the location of the experimentally extracted MAR/SARs reported in the previous study [28] and the MRS predicted MAR/SARs. A genomic map was constructed to illustrate the position of the chromosome breaks relative to the location of the MAR/SARs.

### Quantification of gene cleavage frequency

One to two sets of nested IPCR assays were performed for each experiment. Each set of IPCR assay consisted of five to eight IPCR replicates per cell sample. The number of IPCR bands which represent the DNA fragments derived from the cleaved *AF9* SAR and non-SAR regions was counted. Gene cleavage frequency expresses the median number of chromosome breaks detected within the *AF9* SAR region or non-SAR region in two to three independent experiments.

### Statistical analysis

The Student’s *t* test was used to assess the difference between untreated control and treated samples in the detections of PS externalisation and MMP disruption. The Mann–Whitney *U* test was used to analyse the difference between untreated control and treated samples in the nested IPCR assays. For the detections of PS externalisation and MMP disruption, data are presented as means and standard deviation (SD). For the IPCR assays, data are expressed as median and interquartile range (IQR). Differences were considered statistically significant at *p* value < 0.05. All statistical tests are two-sided.

## Results

### In silico prediction of MAR/SAR

By using MRS, 29 possible MAR/SAR sites were predicted in the *AF9* gene. The nucleotide positions of the MRSs with their sequence composition, relative orientation, distance between the two sequence elements and location of the MRSs in the exon or intron of the *AF9* gene are shown in Table 1. Out of the 29 predicted MAR/SAR sites, 14 were found in intron 2 (MAR/SARs 2–15 in Table 1). Intron 2 is the largest intron of the *AF9* gene which is approximately 164 kb in length. Five MAR/SAR sites were predicted in each intron 3b (MAR/SARs 17–21 in Table 1) and intron 4 (MAR/SARs 22–26 in Table 1). Intron 7 was found to contain two potential MAR/SAR sites (MAR/SARs 27–28 in Table 1). One MAR/SAR site was predicted in each intron 1 (MAR/SAR 1 in Table 1), intron 3a (MAR/SAR 16 in Table 1) and intron 9 (MAR/SAR 29 in Table 1).

**Table 1:** MAR/SARs predicted within the *AF9* gene by using MRS

Predicted MAR/SAR	AWWRTAANNWWGNNNC (16 bp)	Nucleotide position	AATAAYAA (8 bp)	Nucleotide position	Distance (bp)	Location in exon/intron
1	ATAATAATAAAAGCCC (C)	916–931	AATAATAA (C) AATAATAA (C)	915–922 918–925	Overlap Overlap	Intron 1
2	ATAGTAAGGATGGCTG (W)	5636–5651	AATAATAA (W)	5694–5701	42	Intron 2
3	AAAATAACAAAGGAAG (W)	10,555–10,570	AATAACAA (W)	10,561–10,568	Overlap	Intron 2
4	AATATTATTATGGGTC (W)	26,366–26,381	AATAATAA (W)	26,420–26,427	38	Intron 2
5	AAAGTAAACTGGAAAC (C)	47,851–47,866	AATAACAA (W)	47,627–47,634	− 216	Intron 2
6	AAAATAATAATAATAC (W)	56,224–56,239	AATAATAA (W) AATAATAA (W)	56,227–56,234 56,230–56,237	Overlap Overlap	Intron 2
7	AAAATCATCTTGGGAC (W)	94,045–94,060	AATAACAA (W)	93,895–93,902	− 142	Intron 2
8	AAAATAATAAAAACCC (C)	108,633–108,648	AATAATAA (C)	108,635–108,642	Overlap	Intron 2
9-1	ATAATAACATTTTACC (C)	112,369–112,384	AATAATAA (C)	112,368–112,375	Overlap	Intron 2
9-2	AAAATAATAATTGTAC (C)	113,269–113,284	AATAATAA (C)	113,271–113,278	Overlap	Intron 2
10	ATTGGAATGTAGAAAC (W)	117,722–117,737	AATAACAA (W)	117,606–117,613	− 108	Intron 2
11-1	AATATAATCTAATTGC (W)	128,593–128,608	AATAACAA (C)	128,355–128,362	− 230	Intron 2
11-2	AAAATAAGTTTCCAGC (W)	129,838–129,853	AATAACAA (C) AATAATAA (C)	129,941–129,948 129,980–129,987	87 126	Intron 2
12	ATAATAATAAAATCAC (W)	136,176–136,191	AATAATAA (W)	136,182–136,189	Overlap	Intron 2
13	AATATAATGAATATCC (C)	139,927–139,942	AATAATAA (C) AATAATAA (C) AATAATAA (C) AATAATAA (C)	139,902–139,909 139,905–139,912 139,908–139,915 139,911–139,918	− 17 − 14 − 11 − 8	Intron 2
14	TTTATAAACTTGTTTC (C)	151,672–151,687	AATAATAA (W)	151,857–151,864	169	Intron 2
15	AAAATAAAAAAGAGCT (C)	158,541–158,556	AATAATAA (W)	158,593–158,600	46	Intron 2
16	AAAATAATAAATACGC (W)	170,523–170,538	AATAATAA (W) AATAACAA (W)	170,529–170,536 170,619–170,626	Overlap 80	Intron 3a
17-1	ATAATAAATATGAATA (W)	178,638–178,653	AATAATAA (W)	178,634–178,641	Overlap	Intron 3b
17-2	AAAAGAACTAAGGTAC (W)	179,378–179,393	AATAATAA (W)	179,140–179,147	− 230	Intron 3b
18	AAGGTAAATTAGCAGC (W) ATAATAATAATGTTCT (C) ATAATAATGTTCTACC (C)	182,936–182,951 183,171–183,186 183,174–183,189	AATAATAA (C)	183,173–183,180	221 Overlap Overlap	Intron 3b
19	ATTATAAGAAAAATTC (W) ATAATAAAAATGTTAT (C) ATAATGATCAAGTACC (C)	191,065–191,080 191,076–191,091 191,548–191,563	AATAATAA (W)	191,323–191,330	242 231 − 217	Intron 3b
20-1	AAAATAAGAAAACATC (W) AATATAATTATGCTAA (W)	194,333–194,348 194,753–194,768	AATAATAA (W)	194,511–194,518	162 − 234	Intron 3b
20-2	AATATAAAATTGCAAG (W)	195,275–195,290	AATAATAA (C)	195,198–195,205	− 69	Intron 3b
21	AAAATAATAAAGCCAT (W)	200,768–200,783	AATAATAA (W)	200,774–200,781	Overlap	Intron 3b
22-1	ATAATAATAATAATAC (W)	215,365–215,380	AATAATAA (W) AATAATAA (W) AATAATAA (W)	215,368–215,375 215,371–215,378 215,374–215,381	Overlap Overlap Overlap	Intron 4
22-2	AAAATAAAACTGACTC (C) ATAATTACATAGACAC (W)	215,781–215,796 216,113–216,128	AATAACAA (C)	215,941–215,948	144 − 164	Intron 4
23	ATAATAATAATGAAAG (C)	227,849–227,864	AATAATAA (C) AATAATAA (C)	227,848–227,855 227,851–227,858	Overlap Overlap	Intron 4
24-1	ATAATAAGTTATAGGC (W)	236,299–236,314	AATAATAA (W) AATAATAA (C)	236,308–236,315 236,343–236,350	Overlap 28	Intron 4
24-2	AAAATAACAAAATGTC (W) AATGTAAGCAATATCC (W)	237,605–237,620 237,817–237,832	AATAACAA (W)	237,611–237,618	Overlap − 198	Intron 4
24-3	AATGTAAGCAATATCC (W) AAAGTATTGTAGACCC (C) AAAATAATAAAGGGGT (W)	237,817–237,832 237,983–237,998 237,995–238,010	AATAATAA (W)	238,001–238,008	168 2 Overlap	Intron 4
24-4	TATATAATAAAGTGAC (C)	238,794–238,809	AATAATAA (W)	239,050–239,057	240	Intron 4
25-1	ATAATAATGAAGAAAG (C)	246,610–246,625	AATAATAA (C)	246,588–246,595	− 14	Intron 4
25-2	ATTGTAATATTGATTG (C) AATATTACAATGAATC (W) TTTATAAATTAGGGAC (W)	247,587–247,602 247,582–247,597 247,758–247,773	AATAACAA (C)	247,561–247,568	− 18 − 13 − 189	Intron 4
26-1	AAAGTAAATAAAAAAC (W)	251,265–251,280	AATAATAA (C)	251,344–251,351	63	Intron 4
26-2	AATGAAAGGAAGAGCC (W) ATAATAATAATGAAAA (C)	252,636–252,651 252,891–252,906	AATAATAA (C)	252,893–252,900	241 Overlap	Intron 4
27	ATAATAAACTACCATC (W) ATAATAATAAACTACC (W) AAAATAAACTATTTTC (C)	262,931–262,946 262,934–262,949 263,198–263,213	AATAATAA (W)	262,940–262,947	Overlap Overlap − 250	Intron 7
28-1	AATATAATCTTGAACG (C)	265,612–265,627	AATAATAA (C)	265,768–265,775	140	Intron 7
28-2	AAAATAAAAATATGCC (C) AATAAAAATATGCCCC (C) ATAATAAGGCTGGGAC (C) ATTTTAAGAATGAGTC (W) AAGATAAATTAGGTCC (C)	266,963–266,978 266,965–266,980 267,134–267,149 267,205–267,220 267,317–267,332	AATAATAA (C)	267,133–267,140	154 152 Overlap − 64 − 176	Intron 7
28-3	AAGATAAATTAGGTCC (C)	267,317–267,332	AATAATAA (C)	267,569–267,576	236	Intron 7
29	AAAAAAAAATTGTAAC (W)	273,593–273,608	AATAACAA (W)	273,528–273,535	− 57	Intron 9

Nucleotide positions of the MRSs with their sequence composition, relative orientation (C: Crick strand and W: Watson strand), distance between the two sequence elements and location of the MRSs in the exon or intron of the *AF9* gene are shown. A negative distance indicates that 8 bp sequence element precedes the 16 bp sequence element

**Fig. 1:**
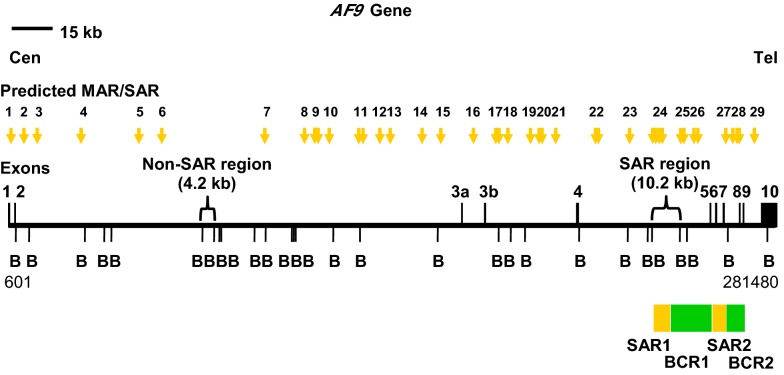
Distribution of potential MAR/SAR sites predicted in the *AF9* gene. The *AF9* genomic map from nucleotide positions 601–281,480 is illustrated above [EMBL:ENSG00000171843]. The locations of exons 1 to 10 and *Bam*H I (B) restriction sites are shown. Green boxes indicate the two patient BCRs reported in the previous study. These two patient BCRs were denominated as BCR1 and BCR2 [28]. Yellow boxes indicate the two MAR/SARs that were biochemically identified by Strissel and co-workers. These two MAR/SARs were designated as SAR1 and SAR2 [28]. Yellow arrows represent the potential MAR/SAR sites predicted by MRS in our study. Clusters of more than one MRS within close proximity are regarded as a single potential MAR/SAR site. Three MRSs were found in SAR1 (MAR 24-2, 24-3, 24-4). One MRS (MAR 27) has been predicted next to the SAR2. Based on the in silico prediction of MAR/SAR, a SAR region (contains MAR/SAR) and a non-SAR region (does not contain MAR/SAR) were determined to be the regions of study

The distribution of the predicted MAR/SAR sites in the *AF9* gene is illustrated in Fig. 1. Based on this in silico prediction of MAR/SAR, we determined a SAR region (contains MAR/SAR) and a non-SAR region (does not contain MAR/SAR) as the targeted regions of our study. The *AF9* SAR region contains four MRSs (MAR/SARs 24-1 to 24-4 in Table 1). However, they are regarded as a single potential MAR/SAR site (MAR/SAR 24) because they were found in close proximity to each other (< 1.5 kb). Three out of these four MRSs were found within the biochemically defined SAR1 (located in intron 4) [28]. On the contrary, the *AF9* non-SAR region is a region which contains neither biochemically defined MAR/SAR nor MRS predicted MAR/SAR.

### Apoptosis detection in H_2_O_2_-treated NP69 and HK1 cells

By using flow cytometric analyses of PS externalisation and MMP disruption, significant percentages of apoptosis were detected in H_2_O_2_-treated NP69 and HK1 cells. These data have been reported in our previous study [26]. Our findings indicate that H_2_O_2_ could induce apoptosis in NP69 and HK1 cells.

### IPCR detection of chromosome breaks mediated by H_2_O_2_-induced apoptosis in NP69 cells

To detect chromosome breaks within the *AF9* SAR and non-SAR regions in cells undergoing H_2_O_2_-induced apoptosis, nested IPCR assay was performed. In the SAR region, IPCR primers were designed to detect chromosome breaks within the first breakpoint cluster region of the *AF9* gene (BCR1). The *AF9* BCR1 is located at the telomeric end of intron 4. It is bordered by two biochemically defined MAR/SARs [27, 28]. The SAR region also contains one MRS predicted MAR/SAR (MAR/SAR 24 in Table 1). The non-SAR region is a region which contains neither biochemically defined MAR/SAR nor MRS predicted MAR/SAR. The intact IPCR band for the *AF9* SAR region and non-SAR region are 944 bp (~ 950 bp) and 956 bp (~ 950 bp), respectively. If there is chromosome break within the region of study, for both SAR and non-SAR regions, IPCR band of less than 950 bp will be detected.

#### AF9 SAR region

**Fig. 2:**
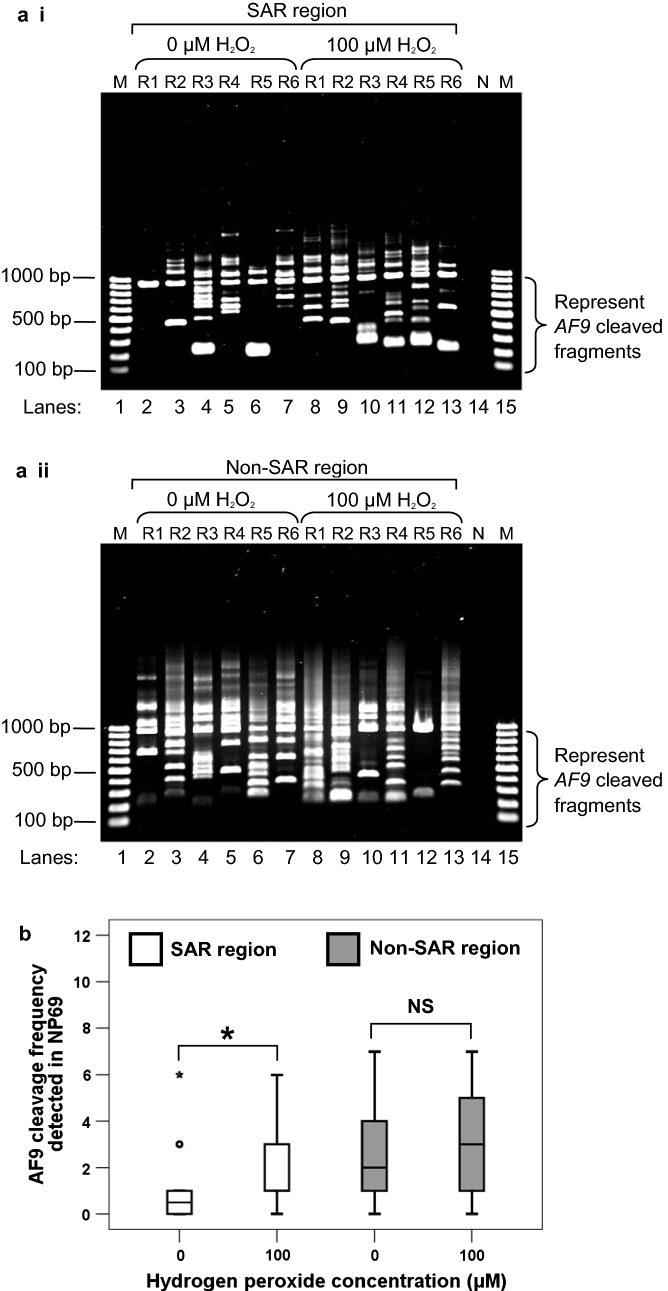
Cleavage frequencies of the *AF9* SAR and non-SAR regions in H_2_O_2_-treated NP69 cells. **a** Representative gel pictures showing the IPCR results of the *AF9* gene in H_2_O_2_-treated NP69 cells: *i* SAR region, *ii* Non-SAR region. NP69 cells were either untreated (lanes 2–7) or treated with 100 µM of H_2_O_2_ for 16 h (lanes 8–13). The cells were harvested for gDNA extraction and nested IPCR. For each cell sample, six IPCR replicates (R1–R6) were prepared. The side brackets show the IPCR bands derived from the cleavages of the *AF9* gene. M: 100 bp DNA ladder. N: Negative control for IPCR. **b** *AF9* cleavage frequency detected in NP69 cells. Data are representative of three independent experiments. Each experiment consisted of 1–2 sets of IPCR. Each set of IPCR was performed in 5–8 IPCR replicates per cell sample. The results are presented as medians with IQRs. *P < 0.05; NS: no significant difference (Mann–Whitney *U* test)

As shown in Fig. 2a i, numerous IPCR bands smaller than 950 bp which represent the cleaved *AF9* gene were identified in NP69 cells treated with H_2_O_2_ (lanes 8–13). The untreated NP69 cells also show a few IPCR bands (lanes 3–7). By using flow cytometric analysis of phosphatidylserine (PS) externalisation, we detected a small amount of apoptotic cells in the untreated sample ([26], Fig. 1). These apoptotic cells might undergo spontaneous DNA breaks and contribute to the background as seen in lanes 3–7. As summarised by the box plot in Fig. 2b, the median *AF9* cleavage frequency of H_2_O_2_-treated NP69 cells was 2.0-fold higher than that of the untreated control cells (*p* = 0.008). Our findings clearly indicate that H_2_O_2_-induced apoptosis results in cleavages within the *AF9* SAR region.

#### AF9 non-SAR region

As shown in Fig. 2a ii, numerous IPCR bands of less than 950 bp which represent the cleaved *AF9* gene were detected in both untreated NP69 cells (lanes 2–7) and NP69 cells treated with H_2_O_2_ (lanes 8–13). However, there was no significant difference between the untreated cells and H_2_O_2_-treated cells in the cleavage frequency of the *AF9* non-SAR region (*p* = 0.739) (Fig. 2b).

### IPCR detection of chromosome breaks mediated by H_2_O_2_-induced apoptosis in HK1 cells

#### AF9 SAR region

**Fig. 3:**
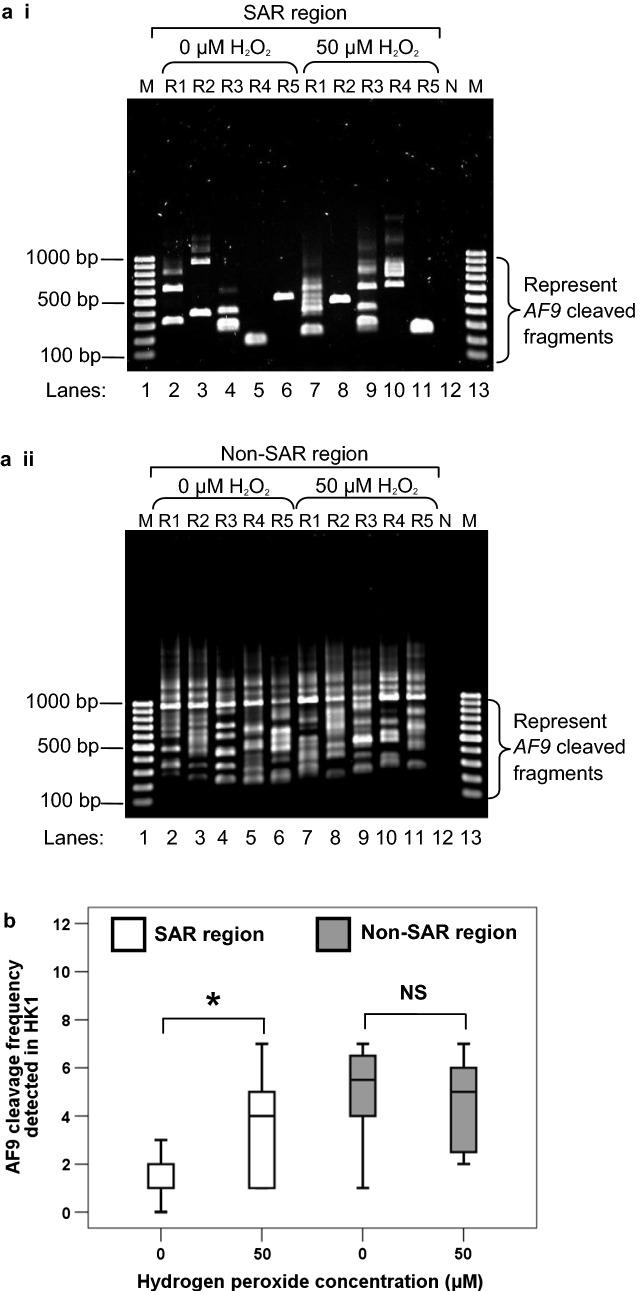
Cleavage frequencies of the *AF9* SAR and non-SAR regions in H_2_O_2_-treated HK1 cells. **a** Representative gel pictures showing the IPCR results of the *AF9* gene in H_2_O_2_-treated HK1 cells: *i* SAR region, *ii* non-SAR region. HK1 cells were either untreated (lanes 2–6) or treated with 50 µM of H_2_O_2_ for 8 h (lanes 7–11). The cells were harvested for gDNA extraction and nested IPCR. For each cell sample, five IPCR replicates (R1–R5) were prepared. The side brackets show the IPCR bands derived from the cleavages of the *AF9* gene. M: 100 bp DNA ladder. N: Negative control for IPCR. **b** *AF9* cleavage frequency detected in HK1 cells. Data are representative of two independent experiments. Each experiment consisted of 1–2 sets of IPCR. Each set of IPCR was performed in 5–6 IPCR replicates per cell sample. The results are expressed as medians with IQRs. *P < 0.05; NS: no significant difference (Mann–Whitney *U* test)

To further strengthen our observation that H_2_O_2_ could induce chromosome breaks within the *AF9* SAR region, IPCR detection of chromosome breaks was also performed in H_2_O_2_-treated HK1 cells. The representative gel picture in Fig. 3a i shows that more IPCR bands were identified in H_2_O_2_-treated HK1 cells (lanes 7–11) as compared with the untreated control cells (lanes 2–6). The median *AF9* cleavage frequency of H_2_O_2_-treated HK1 cells was 4.0-fold higher than that of the untreated control cells (*p* < 0.001) (Fig. 3b). These findings strengthen the suggestion that oxidative stress-induced apoptosis leads to *AF9* gene cleavages within the SAR region.

#### AF9 non-SAR region

As shown in Fig. 3a ii, numerous IPCR bands of less than 950 bp which represent the cleaved *AF9* gene were detected in both untreated HK1 cells (lanes 2–6) and H_2_O_2_-treated HK1 cells (lanes 7–11). However, there was no significant difference between the untreated HK1 cells and H_2_O_2_-treated HK1 cells in the cleavage frequency of the *AF9* non-SAR region (*p* = 0.405) (Fig. 3b). Taken together, our findings suggest that MAR/SAR sequence plays a crucial role in defining the location of chromosome breaks in H_2_O_2_-induced apoptosis.

In this study, we hypothesise that MAR/SAR is a preferential site of chromosome breaks. Therefore, less or no chromosome break was expected to be detected in this non-SAR region after H_2_O_2_ treatment. However, the current results are not as what was expected. There are obviously more cleavage bands detected in the non-SAR region compared with the SAR region (Figs. 2 and 3). The box plot in Fig. 2b shows that in the untreated NP69 cells, the median cleavage frequency of the non-SAR region was 4.0-fold higher than that of the SAR region (*p* = 0.002). Similarly, in the untreated HK1 cells, the median cleavage frequency of the non-SAR region was 5.5-fold higher than that of the SAR region (*p* < 0.001) (Fig. 3b). Such a difference might reflect that there are other chromatin structures which may also contribute to DNA fragility. In addition to MAR/SAR sequence, repeat elements have also been well implicated in mediating chromosome breaks [27, 34]. Hence, this prompted us to investigate the possibility of repeat elements in contributing to DNA fragility of the *AF9* non-SAR region.

### Identification of repeat elements within the *AF9* gene

**Fig. 4:**
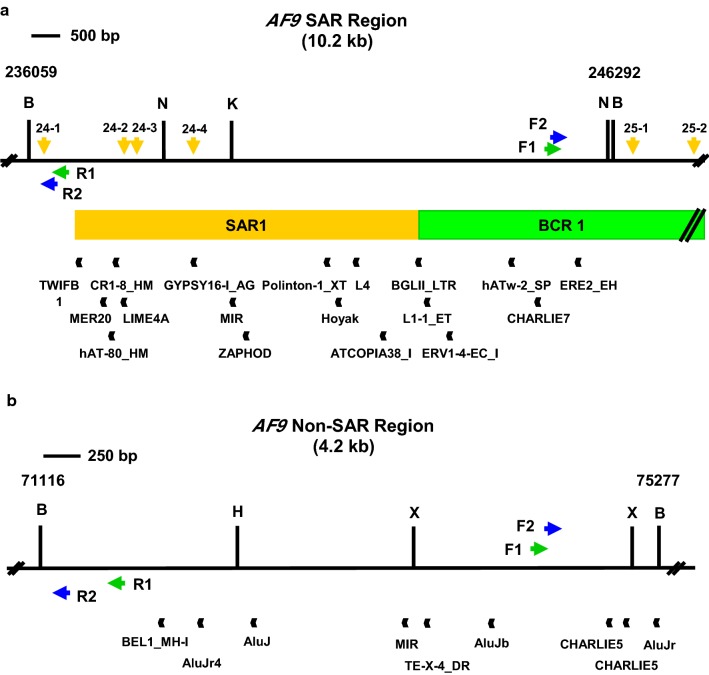
The repeat elements identified within the *AF9* SAR and the non-SAR regions. **a** The SAR region. The SAR region which is bordered by two *Bam*H I sites is 10.2 kb in length (from coordinates 236,059 to 246,292). Green box represents the previously identified patient BCR which is indicated as BCR1. Yellow box shows the previously experimentally isolated MAR/SAR which is indicated as SAR1 [28]. Yellow arrows represent the potential MAR/SAR sites predicted by MRS in the present study. Green and blue arrows represent the primers used in the first and second rounds of nested IPCR, respectively. Black boxes show the repeat elements predicted by CENSOR program. *Bam*H I (B), *Kpn* I (K) and *Nde* I (N) restriction sites are shown. **b** The non-SAR region. The non-SAR region which is bordered by two *Bam*H I sites is 4.2 kb in length (from coordinates 71,116 to 75,277). Green and blue arrows represent the primers used in the first and second rounds of nested IPCR, respectively. Black boxes represent the repeat elements identified by using CENSOR program. *Bam*H I (B), *Hin*d III (H) and *Xba* I (X) restriction sites are shown

CENSOR program (http://www.girinst.org/censor/) was used to identify repeat elements in the *AF9* gene. The repeat elements identified within the SAR and non-SAR regions are shown in Table 2. The locations of repeat elements identified within the SAR and non-SAR regions are illustrated in Fig. 4. There are 18 repeat elements identified within the 10.2 kb SAR region (Table 2). Only one out of these 18 repeat elements is located within the amplified region. The region amplified by the reverse primer (AF9 236211 R) is from coordinates 236,059 to 236,211. This region does not contain any repeat element. The region amplified by the forward primer (AF9 245507 F) is from coordinates 245,507 to 246,292. ERE2_EH (coordinates 245,627–245,728, 102 bp in length) is the only one repeat element identified in this region. It occupies 11% (102 bp) of the amplified SAR region (944 bp).

**Table 2:** Repeat elements in the *AF9* SAR and non-SAR regions predicted by CENSOR program

*AF9* regions	Nucleotide position		Predicted repeat elements	
	From	To	Name	Class
SAR region	236,920	236,987	TWIFB1	DNA/hAT
	237,423	237,476	MER20	DNA/hAT
	237,491	237,548	hAT-80_HM	DNA/hAT
	237,594	237,636	CR1-8_HM	NonLTR/CR1
	237,637	237,719	L1ME4A	NonLTR/L1
	238,883	238,925	GYPSY16-I_AG	LTR/Gypsy
	239,516	239,716	MIR	NonLTR/SINE
	239,786	239,871	ZAPHOD	DNA
	241,267	241,318	Polinton-1_XT	DNA/Polinton
	241,475	241,555	Hoyak1	DNA/hAT
	241,769	241,847	L4	NonLTR/RTEX
	242,176	242,276	ATCOPIA38_I	LTR/Copia
	242,849	242,893	BGLII_LTR	ERV/ERV2
	242,989	243,024	L1-1_ET	NonLTR/L1
	243,397	243,483	ERV1-4-EC_I	ERV/ERV1
	244,480	244,530	hATw-2_SP	DNA/hAT
	244,901	245,043	CHARLIE7	DNA/hAT
	245,627	245,728	ERE2_EH	Interspersed_Repeat
Non-SAR region	71,936	71,999	BEL1_MH-I	LTR/BEL
	72,081	72,368	AluJr4	NonLTR/SINE/SINE1
	72,447	72,695	AluJ	Interspersed_Repeat
	73,459	73,707	MIR	NonLTR/SINE
	73,708	73,761	TE-X-4_DR	Interspersed_Repeat
	74,030	74,304	AluJb	NonLTR/SINE/SINE1
	74,895	74,998	CHARLIE5	DNA/hAT
	75,006	75,169	CHARLIE5	DNA/hAT
	75,192	75,466	AluJr	NonLTR/SINE/SINE1

The *AF9* SAR region is located at coordinates 236,059 to 246,292 [Ensembl:ENSG00000171843]. The nucleotide position, name and class of the predicted repeat elements are shown. The region amplified by the reverse primer (AF9 236,211 R) is from coordinates 236,059 to 236,211 while the region amplified by the forward primer (AF9 245,507 F) is from coordinates 245,507 to 246,292. The amplified SAR region contains one repeat element, namely ERE2_EH (at coordinates 245,627–245,728). The *AF9* non-SAR region is located at coordinates 71,116 to 75,277 [Ensembl:ENSG00000171843]. The nucleotide position, name and class of the predicted repeat elements are shown. The region amplified by the reverse primer (AF9 71,282 R) is from coordinates 71,116 to 71,282 while the region amplified by the forward primer (AF9 74,494 F) is from coordinates 74,494 to 75,277. The amplified non-SAR region contains three repeat elements, namely two CHARLIE5 (at coordinates 74,895–74,998 and 75,006–75,169) and one AluJr (at coordinates 75,192–75,466)

On the other hand, there are nine repeat elements identified within the 4.2 kb non-SAR region (Table 2). Three out of these nine repeat elements are located within the amplified region. The region amplified by the reverse primer (AF9 71282 R) is from coordinates 71,116 to 71,282. There was no repeat element identified in this region. The region amplified by the forward primer (AF9 74494 F) is from coordinates 74,494 to 75,277. There are three repeat elements located in this region, namely two CHARLIE5 (coordinates 74,895–74,998, 104 bp in length and coordinates 75,006–75,169, 164 bp in length) and one AluJr (coordinates 75,192–75,466, 275 bp in length). These three repeat elements (the first CHARLIE5, 104 bp; the second CHARLIE5, 164 bp and AluJr, 275 bp) occupy 57% (543 bp) of the amplified non-SAR region (956 bp). In brief, given that there is no significant difference in the cleavage frequencies between the untreated and H_2_O_2_-treated cells, the chromosome breaks in the non-SAR region were most likely not mediated by H_2_O_2_-induced apoptosis. It is most likely that the presence of the repeat elements contribute to the DNA fragility of the non-SAR region.

### Inhibition of caspase

#### SAR region

**Fig. 5:**
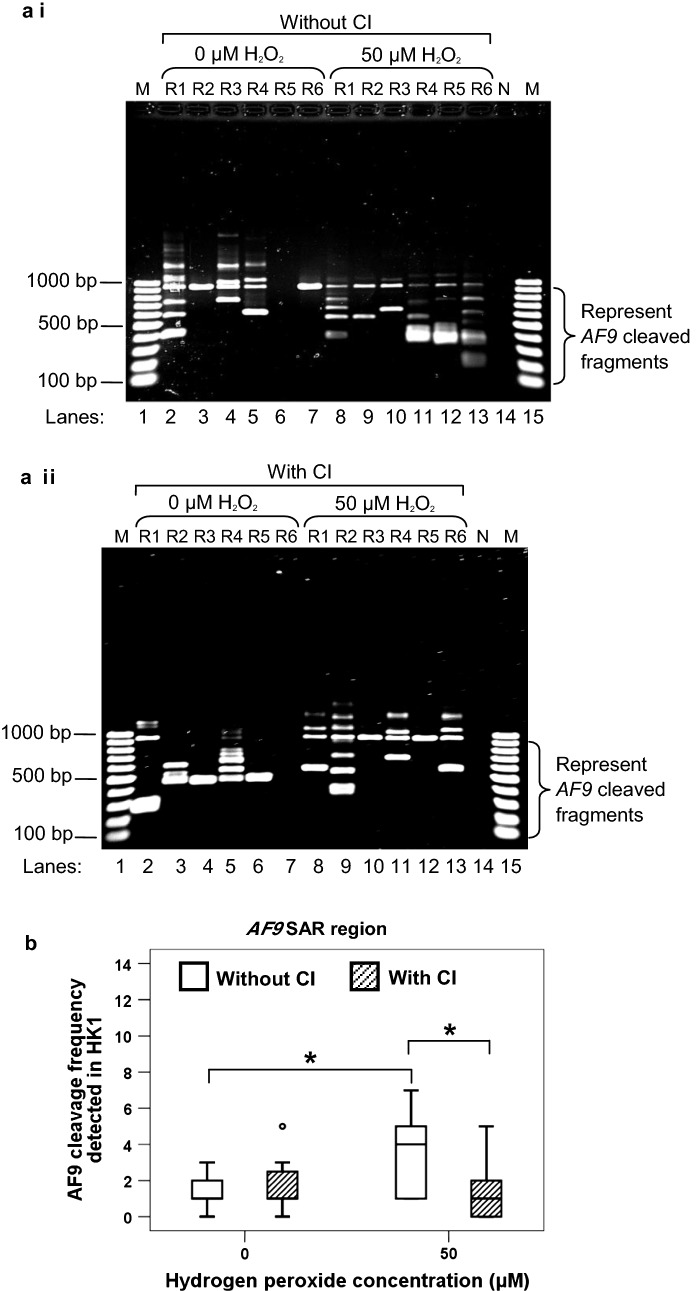
Caspase-3 inhibitor abolishes H_2_O_2_-induced cleavages within the *AF9* SAR region. **a** Representative gel pictures showing the IPCR analysis of the *AF9* SAR region in H_2_O_2_-treated HK1 cells: *i* without CI pre-treatment *ii* with CI pre-treatment. HK1 cells were left untreated or pre-treated with 50 µM of Z-DEVD-FMK for 1 h. The cells were then either untreated (lanes 2–7) or treated with 50 µM of H_2_O_2_ for 8 h (lanes 8–13). Genomic DNA was extracted and modified for nested IPCR. For each cell sample, six IPCR replicates (R1–R6) were prepared. The side brackets show the IPCR bands derived from the cleavages of the *AF9* gene. M: 100 bp DNA ladder. N: Negative control for IPCR. **b** Cleavage frequency of the *AF9* SAR region detected in HK1 cells. Data are representative of two independent experiments. Each experiment consisted of 1–2 sets of IPCR. Each set of IPCR was performed in 5–6 IPCR replicates per cell sample. The results are expressed as medians with IQRs. *P < 0.05 (Mann–Whitney *U* test)

Figure 5a i, ii show the representative IPCR results of the *AF9* SAR region in H_2_O_2_-treated HK1 cells without and with caspase inhibitor (CI) pretreatment, respectively. In the absence of CI, the median cleavage frequency of the *AF9* gene detected in H_2_O_2_-treated HK1 cells was 4.0-fold higher than that of the untreated control cells (*p* < 0.001) (Fig. 5b). The median cleavage frequency of the *AF9* SAR region in H_2_O_2_-treated HK1 cells with CI pre-treatment was 4.0-fold lower than that without CI pre-treatment (*p* = 0.004) (Fig. 5b). These results indicate that H_2_O_2_ induces cleavages within the *AF9* SAR region in a caspase-3-dependent manner.

#### Non-SAR region

**Fig. 6:**
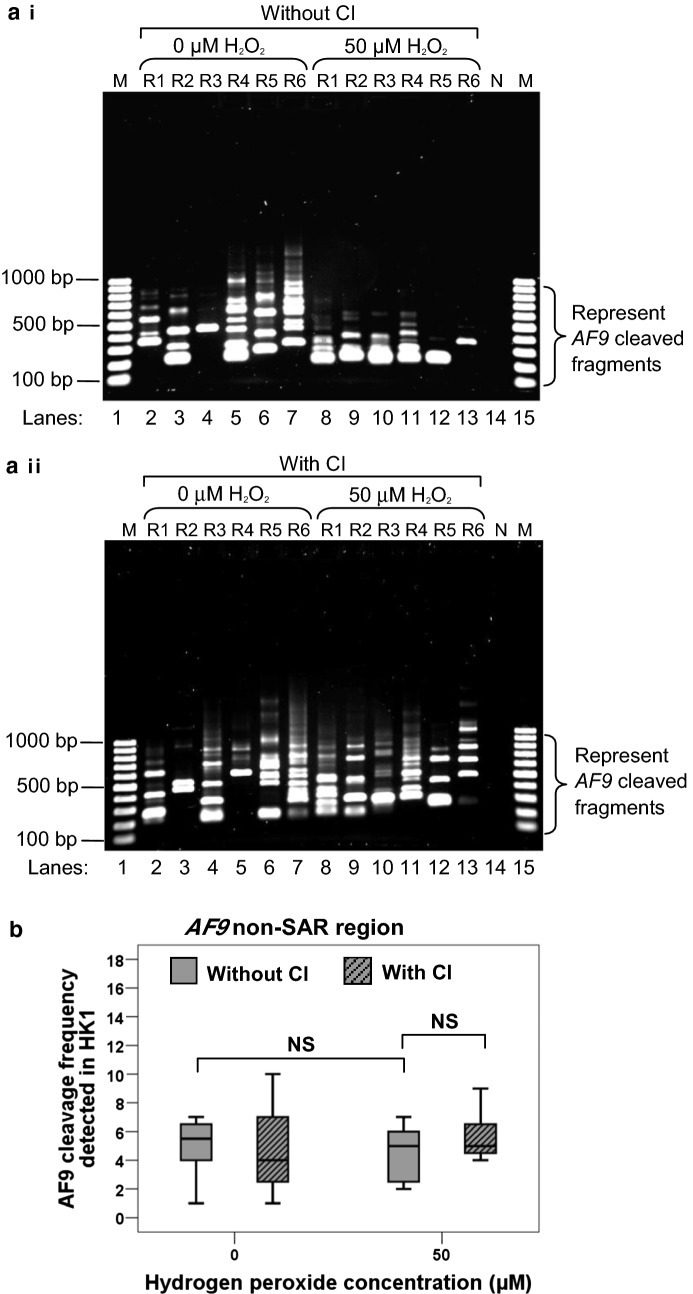
Caspase-3 inhibitor shows no effect on reducing cleavages within the *AF9* non-SAR region. **a** Representative gel pictures showing the IPCR analysis of the *AF9* non-SAR region in H_2_O_2_-treated HK1 cells: *i* without CI pre-treatment *ii* with CI pre-treatment. HK1 cells were left untreated or pre-treated with 50 µM of Z-DEVD-FMK for 1 h. The cells were then either untreated (lanes 2–7) or treated with 50 µM of H_2_O_2_ for 8 h (lanes 8–13). Genomic DNA was extracted and modified for nested IPCR. For each cell sample, six IPCR replicates (R1–R6) were prepared. The side brackets show the IPCR bands derived from the cleavages of the *AF9* gene. M: 100 bp DNA ladder. N: Negative control for IPCR. **b** Cleavage frequency of the *AF9* non-SAR region detected in HK1 cells. Data are representative of two independent experiments. Each experiment consisted of 5–7 IPCR replicates per cell sample. The results are expressed as medians with IQRs. NS: No significant difference (Mann–Whitney *U* test)

Figure 6a i, ii show the representative IPCR results of the *AF9* non-SAR region in H_2_O_2_-treated HK1 cells without and with CI pre-treatment, respectively. There is no significant difference in the cleavage frequency of the non-SAR region between the untreated control and H_2_O_2_-treated HK1 cells (*p* = 0.405) (Fig. 6b). There is also no significant difference in the cleavage frequency between the H_2_O_2_-treated HK1 cells without CI pre-treatment and that with CI pre-treatment (*p* = 0.390) (Fig. 6b). These findings show that CI has no significant effect on the cleavage frequency within the *AF9* non-SAR region. This implies that the cleavages of the non-SAR region are not dependent on caspase-3.

### Sequencing results

**Fig. 7:**
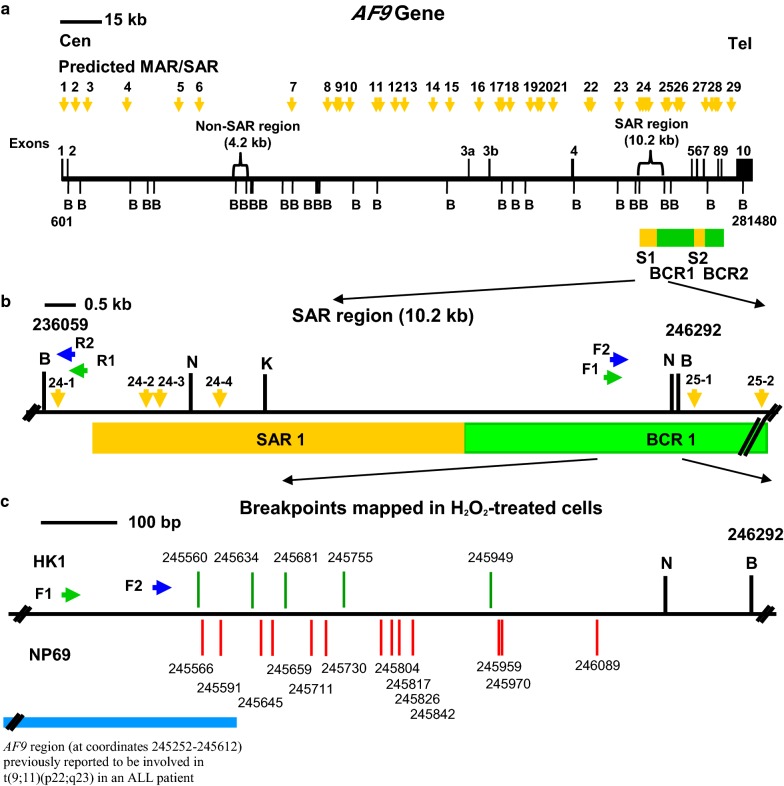
The positions of H_2_O_2_-induced chromosome breaks within the *AF9* SAR region. **a** The *AF9* genomic map from nucleotide positions 601–281,480 [EMBL:ENSG00000171843] [26]. Black vertical lines represent the locations of exons 1–10. Green boxes show the two previously identified patient BCRs, namely BCR1 and BCR2 [28]. Yellow boxes show the two MAR/SARs which were extracted experimentally in the previous study. These two MAR/SARs were indicated as SAR1 and SAR2 [28]. *Bam*H I (B) restriction sites are shown. Yellow arrows represent the potential MAR/SAR sites predicted by MRS in our study. **b** The *AF9* SAR region (10.2 kb). *Bam*H I (B), *Kpn* I (K) and *Nde* I (N) restriction sites are shown. Green and blue arrows represent the primers used in the first and second rounds of nested IPCR, respectively. **c** Breakpoints mapped in H_2_O_2_-treated cells. Red and green vertical lines show the breakpoints identified in H_2_O_2_-treated NP69 and HK1 cells, respectively. All the chromosome breaks were mapped within BCR1 which is bordered SAR1 and SAR2. Blue box represents the *AF9* region (at coordinates 245,252–245,612) that was previously reported to translocate with the *MLL* gene resulting in the formation of the *MLL*-*AF9* fusion gene in an ALL patient [GenBank:AM050804]

Some of the IPCR bands detected within the *AF9* SAR region were excised, purified and sequenced. The sequencing results show that these fragments were all derived from the cleaved *AF9* gene (Additional file 3). Table 3 shows the position of chromosome breaks identified within the *AF9* SAR region in H_2_O_2_-treated NP69 and HK1 cells. Intriguingly, three chromosome breaks (at coordinates 245,560, 245,566 and 245,591) are identified within the *AF9* region (at coordinate 245,252–245,612) that was previously reported to translocate with the mixed lineage leukaemia (*MLL*) gene. This reciprocal translocation t(9;11)(p22;q23) resulted in the formation of *MLL*-*AF9* fusion gene in an acute lymphoblastic leukaemia (ALL) patient [GenBank:AM050804]. Seven breakpoints (at coordinates 245,560, 245,566, 245,591, 245,634, 245,645, 245,659 and 245,681) are within a distance of 70 nucleotides from the breakpoint identified in the ALL patient (at coordinate 245,612) [GenBank:AM050804]. A breakpoint mapped at coordinate 245,591 is similar with that identified in cultured normal blood cells treated with etoposide (VP16) (at coordinate 245,593) [35]. A genomic map illustrating the positions of H_2_O_2_-induced chromosome breaks in NP69 and HK1 cells relative to the MAR/SAR sequences within the *AF9* gene is shown in Fig. 7.

**Table 3:** Breakpoints identified within the *AF9* SAR region in H_2_O_2_-treated cells

Cell line treated with H_2_O_2_	Breakpoint	Remarks
NP69	245,566	This chromosome break was detected within the *AF9* region (at coordinates 245,252–245,612) which was previously found to take part in the *MLL*-*AF9* translocation in an ALL patient [GenBank:AM050804]. This breakpoint is 46 nucleotides different from that identified in an ALL patient (at coordinate 245,612) [GenBank:AM050804]
	245,591	This chromosome break was detected within the *AF9* region (at coordinates 245,252–245,612) which was previously found to take part in the *MLL*-*AF9* translocation in an ALL patient [GenBank:AM050804]. This breakpoint is two nucleotides different from that reported in cultured normal blood cells treated with VP16 (at coordinate 245,593) [35] and 21 nucleotides different from that identified in an ALL patient (at coordinate 245,612) [GenBank:AM050804]
	245,645	This breakpoint is 33 nucleotides different from that identified in an ALL patient (at coordinate 245,612) [GenBank:AM050804]
	245,659	This breakpoint is 47 nucleotides different from that identified in an ALL patient (at coordinate 245,612) [GenBank:AM050804]
	245,711	
	245,730	
	245,804	
	245,817	
	245,826	
	245,842	
	245,959	
	245,970	
	246,089	
HK1	245,560	This chromosome break was detected within the *AF9* region (at coordinates 245,252–245,612) which was previously found to take part in the *MLL*-*AF9* translocation in an ALL patient [GenBank:AM050804]. This breakpoint is 52 nucleotides different from that identified in an ALL patient (at coordinate 245,612) [GenBank:AM050804]
	245,634	This breakpoint is 22 nucleotides different from that identified in an ALL patient (at coordinate 245,612) [GenBank:AM050804]
	245,681	This breakpoint is 69 nucleotides different from that identified in an ALL patient (at coordinate 245,612) [GenBank:AM050804]
	245,755	
	245,949	

The nucleotide positions of the chromosome breaks identified within the *AF9* SAR region were mapped according to the *AF9* sequence retrieved from Ensembl database [EMBL:ENSG00000171843]

## Discussion

Much effort had been employed to identify tumor suppressor genes and oncogenes associated with NPC (reviewed in [36, 37]). However, the underlying mechanism of NPC chromosome rearrangement remains elusive. Oxidative stress has been well implicated in carcinogenesis [38]. Most of the aetiological factors of NPC are known to induce oxidative stress [15–20]. In addition, oxidative stress is also a potent apoptotic inducer [39]. Although apoptosis has long been recognised as a programmed cell death process [40], the perception that cells undergoing apoptosis are destined to die has been challenged [41]. It was shown that cells have the potential to recover from the execution phase of apoptosis through DNA repair. However, surviving cells that have undergone compromised DNA repair may carry chromosome rearrangements [41, 42].

In order to test the apoptotic effect of H_2_O_2_ in NP69 and HK1 cells, we analysed the H_2_O_2_-treated NP69 and HK1 cells by flow cytometric analyses of PS externalisation and MMP loss. Our observations showed that H_2_O_2_ could induce apoptosis in both NP69 and HK1 cells. These data have been published in our previous report [26]. Chromosomal breakage resulting from chromosome loop excision is an initial event in both apoptotic DNA fragmentation and chromosome rearrangement. It was found that chromosome breaks tend to fall in certain regions containing specific chromatin structural elements such as MAR/SAR [27, 28]. MAR/SAR sequences possess unwinding properties which facilitate the entry of protein factors involved in apoptosis, replication, transcription and chromosome condensation [43, 44]. The unwinding properties of MAR/SAR sequences also render them to be more susceptible to cleavage [44, 45]. In our previous report, we demonstrated that high cell density and EBV latent membrane protein 1 (*LMP1*) expression triggered apoptosis in NPC cells. This in turn caused cleavages of the *MLL* BCR at the MAR/SAR sequence. These findings implied that MAR/SAR may play an essential role in defining the cleavage sites during high cell density or *LMP1*-induced apoptosis [46]. In this study, we intended to investigate if MAR/SAR is a preferential site of chromosome breaks mediated by oxidative stress-induced apoptosis.

The human *AF9* gene at 9p22 was targeted in this study for two reasons. Firstly, this gene is one of the most common fusion partner genes of the *MLL* gene at 11q23 [28]. The t(9;11)(p22;q23) has been strongly associated with acute myelogenous leukaemia (AML), less common with therapy-related AML (t-AML), with ALL and myelodysplastic syndromes (MDS) [28, 47]. Secondly, 9p22 is a common chromosomal deletion site in NPC [32]. There were two MAR/SARs isolated experimentally in the *AF9* gene. They were designated as SAR1 and SAR2. SAR1 is found in intron 4, whereas SAR2 spans from exons 5 to 7. Two patient breakpoint cluster regions (BCR) have been identified in the *AF9* gene, namely, BCR1 and BCR2. BCR1 is located in intron 4, whereas BCR2 encompasses introns 7 to 8. These two BCRs are bordered by SAR1 and SAR2 [27, 28].

In the present study, in silico prediction of MAR/SAR was performed by using MRS. MRS is a bipartite sequence element that is specific for a large group of MAR/SARs. MRS consists of two individual sequence elements that are approximately 200 bp apart. However, when the DNA is wrapped around the histones, these two sequence elements are located at a position near the dyad axis of the nucleosome. Therefore, they can be aligned together in MAR/SAR after the nucleosomes are positioned. The close proximity between these two sequence elements on the positioned nucleosome enables them to create a protein binding site in MAR/SAR. In the study of van Drunen and co-workers, more than 300 kb of DNA sequence from several eukaryotic organisms were analysed. Their findings showed that all the MRSs that have been identified map specifically to the biochemically identified MAR/SARs [33]. MRS has been widely used in previous studies. MRS has been used to predict MAR/SAR in the human *LMP*/*TAP* gene region. All of the five predicted MAR/SARs in the analysed region match to the experimentally defined MAR/SARs [48]. Besides, MRS has also been used to identify the positions of MAR/SARs in human β-globin locus [49] and wheat high-molecular-weight glutenin *1Dy10* gene promoter [50].

The potential MAR/SAR sites predicted by MRS in the present study were compared with the location of biochemically identified MAR/SAR reported in previous studies [27, 28]. Strissel et al. have analysed 61 kb of the *AF9* region for MAR/SAR. Their region of study encompassed exons 4 to 10. In their region of study, two MAR/SARs were identified through experimental extraction. These two MAR/SARs were designated as SAR1 and SAR2. SAR1 is a 6.2 kb MAR/SAR located in intron 4 whereas SAR2 is a 4.6 kb MAR/SAR spans through parts of introns 5 to 7. To the extent of our knowledge, no analysis on MAR/SAR was reported for the *AF9* region from exon 1 to intron 3.

Within the *AF9* gene of 280,880 bp in length, 29 possible MAR/SAR sites were predicted in our study. Four MRSs (MAR/SARs 24-1 to 24-4 in Table 1 and Fig. 1) are associated with SAR1. However, these four MRSs are regarded as a single potential MAR/SAR site (MAR/SAR 24) because they cluster within close proximity to each other (< 1.5 kb). Three out of these four MRSs fall within SAR1 (MAR/SARs 24-2 to 24-4 in Table 1 and Fig. 1). One of the MRSs was found in a region < 1 kb centromeric to SAR1 (MAR/SARs 24-1 in Table 1 and Fig. 1). In addition, one MAR/SAR site (MAR/SAR 27 in Table 1 and Fig. 1) has been predicted in a region < 1.5 kb telomeric to SAR2.

In the present study, both the normal transformed nasopharyngeal epithelial cells (NP69) and nasopharyngeal carcinoma cells (HK1) were used. In both of these cell lines, oxidative stress-induced apoptosis results in cleavages within the *AF9* SAR sequences. To relate the position of H_2_O_2_-induced chromosome breaks with the MAR/SAR sites, the IPCR bands that represent cleavages within the *AF9* SAR region were sequenced. The sequencing results revealed that all the chromosome breaks were mapped within BCR1 which is bordered by SAR1 and SAR2 (Fig. 7). Intriguingly, a few chromosome breaks were mapped within the region of *AF9* that was previously reported being involved in the formation of the *MLL*-*AF9* fusion gene in an ALL patient [GenBank:AM050804]. Similar chromatin structural elements have been identified in the BCRs of the *AF9* and *MLL* genes. These include DNase I hypersensitive (HS) cleavage sites, MAR/SAR sequences and topoisomerase II cleavage sites. The similarity in the structural elements is suggested to cause the *AF9* and *MLL* BCRs to be the recombination hotspots resulting in *MLL*-*AF9* translocations in leukaemia [28]. Taken together, our results are consistent with those of the other studies which found that MAR/SAR may be a preferential site of chromosome breaks in apoptosis [51] and chromosome rearrangements [27–29]. Considering the observations in leukaemic cells and nasopharyngeal epithelial cells, it is plausible that regardless of the cancer type, the chromatin structure could be playing a vital role in determining the site of chromosome rearrangement.

In order to further examine the involvement of MAR/SAR in stress-induced chromosome breaks formation, the cleavage frequency of the *AF9* SAR region was compared with that of the *AF9* non-SAR region. We hypothesised that MAR/SAR is a preferential site of chromosome breaks, thus we expected to see less or no chromosome breaks detected in the non-SAR region after H_2_O_2_ treatment. However, to our surprise, in both untreated NP69 and HK1 cells, the cleavage frequencies of the non-SAR region were significantly higher than those of the SAR region. These findings imply that there are other chromatin structures which may also contribute to DNA fragility. In addition to MAR/SAR sequence, repeat elements have also been strongly implicated in mediating chromosome breaks [27, 34]. Thus, the possibility of repeat elements in contributing to DNA fragility of the *AF9* non-SAR region was explored.

By using CENSOR program, three repeat elements (the first CHARLIE5, 104 bp; the second CHARLIE5, 164 bp and AluJr, 275 bp) were identified in the amplified non-SAR region of the *AF9* gene. These repeat elements occupy 57% (543 bp) of the amplified non-SAR region (956 bp). On the contrary, ERE2_EH (102 bp in length) is the only one repeat element identified in the amplified SAR region. It occupies 11% (102 bp) of the amplified SAR region (944 bp).

It is noteworthy that, although the cleavage frequency of the non-SAR region detected in the untreated cells was higher than that of the SAR region, there was no significant difference between the H_2_O_2_-treated cells and untreated cells in the cleavage frequency of the *AF9* non-SAR region. This is true for both NP69 and HK1 cells. Hence, it can be suggested that the cleavages identified in the non-SAR region were not stress-induced or stress-mediated. It is likely that the presence of repeat elements renders the chromosome to be more prone to cleavage. Previous studies have reported that common fragile sites, including FRA3B, FRA7G, FRA7H, FRA16D and FRAXB have all been shown to contain a high proportion of repeat elements, such as interspersed repeat elements, long terminal repeats (LTR), transposable elements, Mirs, L1 elements, L2 elements and *Alu* elements. These repetitive elements have been associated with the fragility of these fragile sites [52, 53]. The findings of our study conclude that MAR/SAR may be a preferential site of chromosome breaks during oxidative stress-induced apoptosis and may play an important role in oxidative stress-induced chromosome rearrangement.

We have previously demonstrated that H_2_O_2_ induces apoptosis in NP69 and HK1 cells in a caspase-3-dependent manner. By using Caspase-Glo 3/7, a luminescence-based assay, activation of caspase-3/7 was detected in H_2_O_2_-treated NP69 and HK1 cells. Pretreatment with Z-DEVD-FMK inhibits the activity of caspase-3/7 in H_2_O_2_-treated cells [26]. In the cytoplasm of healthy cells, CAD exists naturally as a heterodimer with its chaperone, inhibitor of CAD (ICAD). ICAD possesses two caspase-3 cleavage sites. Upon caspase-3-mediated cleavage of ICAD, CAD is released from ICAD. Subsequently, CAD enters the nucleus and cleaves DNA by generating double-strand breaks [54, 55]. Given that ICAD is primarily inactivated by DEVD-cleaving caspase-3 [56], inhibiting caspase-3 by using Z-DEVD-FMK is the most effective way of inactivating CAD. Therefore, if CAD is responsible for mediating chromosome breaks in H_2_O_2_-induced apoptosis, the chromosome breaks in H_2_O_2_-treated cells will be reduced or eliminated when caspase-3 is inhibited.

For the *AF9* SAR region, inhibition of caspase by Z-DEVD-FMK significantly reduced the *AF9* cleavages in H_2_O_2_-treated HK1 cells. Our findings suggest that, H_2_O_2_ induces chromosome breaks through caspase-3 activation. This study confirms the claims made in previous researches where H_2_O_2_ induces DNA fragmentation in a caspase-3-dependent manner [39]. Given that activated caspase-3 can stimulate CAD which is responsible for apoptotic DNA fragmentation, CAD is most likely the major player responsible for H_2_O_2_-induced chromosome breaks within the *AF9* SAR region. Indeed, our previous study had demonstrated that, overexpression of ICAD resulted in expression of CAD and also inhibited H_2_O_2_-induced *MLL* gene cleavages. The observations of our previous study suggested a role for CAD in mediating H_2_O_2_-induced chromosome breaks [57].

In addition, our findings were supported by other research that CAD preferentially binds to the nuclear matrix of cells undergoing apoptosis. CAD/ICAD complex is freely moving in dividing cells. However, once apoptosis is induced, the mobility of the activated CAD becomes gradually restricted. The immobilisation of CAD is due to its association with the nuclear matrix [51]. Nuclear matrix is the binding site for the organisation of DNA loop structure [58]. DNA interacts with the nuclear matrix through MAR/SAR sequences [59]. When CAD binds to the nuclear matrix during apoptosis [51], it is in close proximity to the MAR/SAR sequences of the DNA loops. Hence, CAD potentially cleaves the DNA at the MAR/SAR sequences when it is being associated with the nuclear matrix. The reduction of cleavages within the SAR region by inhibiting CAD thus supports our hypothesis that CAD cleaves the DNA preferentially at the MAR/SAR sites during oxidative stress.

By contrast, Z-DEVD-FMK shows no effect on reducing cleavages within the *AF9* non-SAR region. This indicates that the cleavages within the *AF9* non-SAR region are neither dependent on caspase-3 nor CAD. Since H_2_O_2_ induces apoptosis and chromosome breaks in a caspase-3-dependent manner, these findings therefore strengthen the evidence that the cleavages within the *AF9* non-SAR region are not mediated by H_2_O_2_-induced apoptosis.

In the current study, there are some limitations in using in silico prediction of MAR/SAR. The length and exact location of MAR/SAR could not be determined by using MRS-prediction. In order to study MAR/SAR in a more comprehensive way, biochemical isolation of MAR/SAR may be carried out simultaneously with in silico prediction in future works. This may be done by using a Southern blot-based SAR mapping assay [28]. Nevertheless, the positions of MRS-predicted MAR/SAR may serve as a guide for designing suitable probes to identify biochemically isolated MAR/SAR. As for the comparison of SAR region and non-SAR region, more non-SAR regions may be studied. These could help further elucidate the roles of MAR/SAR in stress-induced chromosome breakages and rearrangements.

## Conclusions

Our results clearly demonstrate that oxidative stress-induced apoptosis results in the *AF9* gene cleavages within the region that contains MAR/SAR. This implies that MAR/SAR may play an important role in defining the location of chromosomal cleavages during oxidative stress-induced apoptosis. In addition, the apoptotic nuclease CAD may be closely associated with MAR/SAR in mediating these oxidative stress-induced chromosomal cleavages. By investigating the role of MAR/SAR and its association with CAD, our findings provide deeper insights into the potential role of oxidative stress-induced apoptosis in mediating the chromosome rearrangements in nasopharyngeal epithelial cells.

## Additional files

**Additional file 1.** Description of exons and introns in the *AF9* gene.

**Additional file 2.** DNA manipulation steps in preparation for nested IPCR.

**Additional file 3.** DNA sequencing data.

## Abbreviations

NPC: nasopharyngeal carcinoma
CAD: caspase-activated deoxyribonuclease
MAR/SAR: matrix association region/scaffold attachment region
MRS: MAR/SAR recognition signature
H_2_O_2_: hydrogen peroxide
PS: phosphatidylserine
MMP: mitochondrial membrane potential
IPCR: inverse polymerase chain reaction
BCR: breakpoint cluster region
MLL: mixed lineage leukaemia
ALL: acute lymphoblastic leukaemia
EBV: Epstein–Barr virus
ROS: reactive oxygen species
ICAD: inhibitor of caspase-activated deoxyribonuclease
CPT: camptothecin
VP16: etoposide
PI: propidium iodide
CI: caspase inhibitor

## References

[1] Henle G, Henle W (1976). Epstein–Barr virus-specific IgA serum antibodies as an outstanding feature of nasopharyngeal carcinoma. Int J Cancer.

[2] Raab-Traub N (1992). Epstein–Barr virus and nasopharyngeal carcinoma. Semin Cancer Biol.

[3] Armstrong RW, Armstrong MJ, Yu MC, Henderson BE (1983). Salted fish and inhalants as risk factors for nasopharyngeal carcinoma in Malaysian Chinese. Cancer Res.

[4] Fong LY, Ho JH, Huang DP (1979). Preserved foods as possible cancer hazards: WA rats fed salted fish have mutagenic urine. Int J Cancer.

[5] Huang DP, Ho JH, Saw D, Teoh TB (1978). Carcinoma of the nasal and paranasal regions in rats fed Cantonese salted marine fish. IARC Sci Publ.

[6] Yu MC, Nichols PW, Zou XN, Estes J, Henderson BE (1989). Induction of malignant nasal cavity tumours in Wistar rats fed Chinese salted fish. Br J Cancer.

[7] Fong YY, Chan WC (1977). Nitrate, nitrite, dimethylnitrosamine and *N*-nitrosopyrrolidine in some Chinese food products. Food Cosmet Toxicol.

[8] Poirier S, Ohshima H, de The G, Hubert A, Bourgade MC, Bartsch H (1987). Volatile nitrosamine levels in common foods from Tunisia, south China and Greenland, high-risk areas for nasopharyngeal carcinoma (NPC). Int J Cancer.

[9] Cheng YJ, Hildesheim A, Hsu MM, Chen IH, Brinton LA, Levine PH (1999). Cigarette smoking, alcohol consumption and risk of nasopharyngeal carcinoma in Taiwan. Cancer Causes Control.

[10] Armstrong RW, Imrey PB, Lye MS, Armstrong MJ, Yu MC, Sani S (2000). Nasopharyngeal carcinoma in Malaysian Chinese: occupational exposures to particles, formaldehyde and heat. Int J Epidemiol.

[11] Vaughan TL, Stewart PA, Teschke K, Lynch CF, Swanson GM, Lyon JL (2000). Occupational exposure to formaldehyde and wood dust and nasopharyngeal carcinoma. Occup Environ Med.

[12] Hildesheim A, Dosemeci M, Chan CC, Chen CJ, Cheng YJ, Hsu MM (2001). Occupational exposure to wood, formaldehyde, and solvents and risk of nasopharyngeal carcinoma. Cancer Epidemiol Biomark Prev.

[13] Hung SH, Chen PY, Lin HC, Ting J, Chung SD (2014). Association of rhinosinusitis with nasopharyngeal carcinoma: a population-based study. Laryngoscope.

[14] Tsou YA, Lin CC, Tai CJ, Tsai MH, Tsai TC, Chen CM (2014). Chronic rhinosinusitis and the risk of nasopharyngeal cancer in a Taiwanese health study. Am J Rhinol Allergy.

[15] Ahotupa M, Bussacchini-Griot V, Bereziat JC, Camus AM, Bartsch H (1987). Rapid oxidative stress induced by *N*-nitrosamines. Biochem Biophys Res Commun.

[16] Carnevali S, Petruzzelli S, Longoni B, Vanacore R, Barale R, Cipollini M (2003). Cigarette smoke extract induces oxidative stress and apoptosis in human lung fibroblasts. Am J Physiol Lung Cell Mol Physiol.

[17] Federico A, Morgillo F, Tuccillo C, Ciardiello F, Loguercio C (2007). Chronic inflammation and oxidative stress in human carcinogenesis. Int J Cancer.

[18] Kum C, Kiral F, Sekkin S, Seyrek K, Boyacioglu M (2007). Effects of xylene and formaldehyde inhalations on oxidative stress in adult and developing rats livers. Exp Anim.

[19] Gruhne B, Sompallae R, Marescotti D, Kamranvar SA, Gastaldello S, Masucci MG (2009). The Epstein–Barr virus nuclear antigen-1 promotes genomic instability via induction of reactive oxygen species. Proc Natl Acad Sci USA.

[20] Pylkkanen L, Stockmann-Juvala H, Alenius H, Husgafvel-Pursiainen K, Savolainen K (2009). Wood dusts induce the production of reactive oxygen species and caspase-3 activity in human bronchial epithelial cells. Toxicology.

[21] Huang SY, Fang CY, Tsai CH, Chang Y, Takada K, Hsuc TY (2014). *N*-methyl-*N*′-nitro-*N*-nitrosoguanidine induces and cooperates with 12-*O*-tetradecanoylphorbol-1,3-acetate/sodium butyrate to enhance Epstein–Barr virus reactivation and genome instability in nasopharyngeal carcinoma cells. Chem Biol Interact.

[22] Wu CC, Fang CY, Cheng YJ, Hsu HY, Chou SP, Huang SY (2017). Inhibition of Epstein–Barr virus reactivation by the flavonoid apigenin. J Biomed Sci.

[23] Sallmyr A, Fan J, Rassool FV (2008). Genomic instability in myeloid malignancies: increased reactive oxygen species (ROS), DNA double strand breaks (DSBs) and error-prone repair. Cancer Lett.

[24] Ma N, Kawanishi M, Hiraku Y, Murata M, Huang GW, Huang Y (2008). Reactive nitrogen species-dependent DNA damage in EBV-associated nasopharyngeal carcinoma: the relation to STAT3 activation and EGFR expression. Int J Cancer.

[25] Zhao F, Wang Q (2012). The protective effect of peroxiredoxin II on oxidative stress induced apoptosis in pancreatic beta-cells. Cell Biosci.

[26] Tan SN, Sim SP, Khoo AS (2016). Potential role of oxidative stress-induced apoptosis in mediating chromosomal rearrangements in nasopharyngeal carcinoma. Cell Biosci.

[27] Strick R, Zhang Y, Emmanuel N, Strissel PL (2006). Common chromatin structures at breakpoint cluster regions may lead to chromosomal translocations found in chronic and acute leukemias. Hum Genet.

[28] Strissel PL, Strick R, Tomek RJ, Roe BA, Rowley JD, Zeleznik L (2000). DNA structural properties of AF9 are similar to MLL and could act as recombination hot spots resulting in MLL/AF9 translocations and leukemogenesis. Hum Mol Genet.

[29] Broeker PL, Super HG, Thirman MJ, Pomykala H, Yonebayashi Y, Tanabe S (1996). Distribution of 11q23 breakpoints within the MLL breakpoint cluster region in de novo acute leukemia and in treatment-related acute myeloid leukemia: correlation with scaffold attachment regions and topoisomerase II consensus binding sites. Blood.

[30] Oberhammer F, Wilson JW, Dive C, Morris ID, Hickman JA, Wakeling AE (1993). Apoptotic death in epithelial cells: cleavage of DNA to 300 and/or 50 kb fragments prior to or in the absence of internucleosomal fragmentation. EMBO J.

[31] Berezney R, Mortillaro MJ, Ma H, Wei X, Samarabandu J (1995). The nuclear matrix: a structural milieu for genomic function. Int Rev Cytol.

[32] Shao JY, Wang HY, Huang XM, Feng QS, Huang P, Feng BJ (2000). Genome-wide allelotype analysis of sporadic primary nasopharyngeal carcinoma from southern China. Int J Oncol.

[33] van Drunen CM, Sewalt RG, Oosterling RW, Weisbeek PJ, Smeekens SC, van Driel R (1999). A bipartite sequence element associated with matrix/scaffold attachment regions. Nucleic Acids Res.

[34] Jeffs AR, Benjes SM, Smith TL, Sowerby SJ, Morris CM (1998). The BCR gene recombines preferentially with Alu elements in complex BCR-ABL translocations of chronic myeloid leukaemia. Hum Mol Genet.

[35] Cynthia PN, Sim SP (2012). Etoposide-induced apoptosis results in chromosome breaks within the *AF*9 gene: its implication in chromosome rearrangement in leukaemia. Adv Biosci Biotechnol.

[36] Lo KW, Huang DP (2002). Genetic and epigenetic changes in nasopharyngeal carcinoma. Semin Cancer Biol.

[37] Liu MJ, Wang XY, Peng Y, Shen SR, Li GY (2014). Egr-1 regulates the transcription of NGX6 gene through a Sp1/Egr-1 overlapping site in the promoter. BMC Mol Biol.

[38] Wiseman H, Halliwell B (1996). Damage to DNA by reactive oxygen and nitrogen species: role in inflammatory disease and progression to cancer. Biochem J.

[39] Kim DK, Cho ES, Um HD (2000). Caspase-dependent and -independent events in apoptosis induced by hydrogen peroxide. Exp Cell Res.

[40] Kerr JF, Wyllie AH, Currie AR (1972). Apoptosis: a basic biological phenomenon with wide-ranging implications in tissue kinetics. Br J Cancer.

[41] Vaughan AT, Betti CJ, Villalobos MJ (2002). Surviving apoptosis. Apoptosis.

[42] Betti CJ, Villalobos MJ, Diaz MO, Vaughan AT (2001). Apoptotic triggers initiate translocations within the MLL gene involving the nonhomologous end joining repair system. Cancer Res.

[43] Gohring F, Schwab BL, Nicotera P, Leist M, Fackelmayer FO (1997). The novel SAR-binding domain of scaffold attachment factor A (SAF-A) is a target in apoptotic nuclear breakdown. EMBO J.

[44] Bode J, Benham C, Ernst E, Knopp A, Marschalek R, Strick R (2000). Fatal connections: when DNA ends meet on the nuclear matrix. J Cell Biochem.

[45] Legault J, Tremblay A, Mirault ME (1997). Preferential localization of DNA damage induced by depurination and bleomycin in a plasmid containing a scaffold-associated region. Biochem Cell Biol.

[46] Yee PH, Sim SP (2010). High cell density and latent membrane protein 1 expression induce cleavage of the mixed lineage leukemia gene at 11q23 in nasopharyngeal carcinoma cell line. J Biomed Sci.

[47] Swansbury GJ, Slater R, Bain BJ, Moorman AV, Secker-Walker LM (1998). Hematological malignancies with t(9;11)(p21–22;q23)—a laboratory and clinical study of 125 cases. European 11q23 Workshop participants. Leukemia.

[48] Donev R, Horton R, Beck S, Doneva T, Vatcheva R, Bowen WR (2003). Recruitment of heterogeneous nuclear ribonucleoprotein A1 in vivo to the LMP/TAP region of the major histocompatibility complex. J Biol Chem.

[49] Ostermeier GC, Liu Z, Martins RP, Bharadwaj RR, Ellis J, Draghici S (2003). Nuclear matrix association of the human b-globin locus utilizing a novel approach to quantitative real-time PCR. Nucleic Acids Res.

[50] Thilmony R, Guttman ME, Lin JW, Blechl AE (2014). The wheat HMW-glutenin *1Dy10* gene promoter controls endosperm expression in *Brachypodium distachyon*. GM Crops Food.

[51] Lechardeur D, Xu M, Lukacs GL (2004). Contrasting nuclear dynamics of the caspase-activated DNase (CAD) in dividing and apoptotic cells. J Cell Biol.

[52] Mimori K, Druck T, Inoue H, Alder H, Berk L, Mori M (1999). Cancer-specific chromosome alterations in the constitutive fragile region FRA3B. Proc Natl Acad Sci USA.

[53] Arlt MF, Miller DE, Beer DG, Glover TW (2002). Molecular characterization of FRAXB and comparative common fragile site instability in cancer cells. Genes Chromosom Cancer.

[54] Enari M, Sakahira H, Yokoyama H, Okawa K, Iwamatsu A, Nagata S (1998). A caspase-activated DNase that degrades DNA during apoptosis, and its inhibitor ICAD. Nature.

[55] Sakahira H, Enari M, Nagata S (1998). Cleavage of CAD inhibitor in CAD activation and DNA degradation during apoptosis. Nature.

[56] Wolf BB, Schuler M, Echeverri F, Green DR (1999). Caspase-3 is the primary activator of apoptotic DNA fragmentation via DNA fragmentation factor-45/inhibitor of caspase-activated DNase inactivation. J Biol Chem.

[57] Boon SS, Sim SP (2015). Inhibitor of caspase-activated DNase expression enhances caspase-activated DNase expression and inhibits oxidative stress-induced chromosome breaks at the mixed lineage leukaemia gene in nasopharyngeal carcinoma cells. Cancer Cell Int.

[58] Mirkovitch J, Gasser SM, Laemmli UK (1988). Scaffold attachment of DNA loops in metaphase chromosomes. J Mol Biol.

[59] Cockerill PN, Garrard WT (1986). Chromosomal loop anchorage of the kappa immunoglobulin gene occurs next to the enhancer in a region containing topoisomerase II sites. Cell.

