# Supplementary material for: Matrix association region/scaffold attachment region (MAR/SAR) sequence: its vital role in mediating chromosome breakages in nasopharyngeal epithelial cells via oxidative stress-induced apoptosis
Source: BMC Mol Biol. 2018 Dec 4;19:15. doi: 10.1186/s12867-018-0116-5 (PMC6278157; doi:10.1186/s12867-018-0116-5)
Supplement: Supplementary file 1 — Additional file 1. Description of exons and introns in the AF9 gene. [file 12867_2018_116_MOESM1_ESM.pdf]

Additional file 1

Description of exons and introns in the *AF9* gene

Exon/Intron	*Nucleotide position*	*Length (bp)*
5’ upstream sequence	1-600	600
Exon 1	601-899	299
Intron 1	900-2309	1410
Exon 2	2310-2490	181
Intron 2	2491-166358	163868
Exon 3a	166359-166441	83
Intron 3a	166442-174878	8437
Exon 3b	174879-175022	144
Intron 3b	175023-208719	33697
Exon 4	208720-209424	705
Intron 4	209425-257400	47976
Exon 5	257401-257476	76
Intron 5	257477-259539	2063
Exon 6	259540-259669	130
Intron 6	259670-262303	2634
Exon 7	262304-262403	100
Intron 7	262404-268265	5862
Exon 8	268266-268337	72
Intron 8	268338-269548	1211
Exon 9	269549-269620	72
Intron 9	269621-276570	6950
Exon 10	276571-281480	4910
3’ downstream sequence	281481-282080	600

The *AF9* gene located at 9p22. It is 280880 bp in length [EMBL:ENSG00000171843].
