# Supplementary material for: Matrix association region/scaffold attachment region (MAR/SAR) sequence: its vital role in mediating chromosome breakages in nasopharyngeal epithelial cells via oxidative stress-induced apoptosis
Source: BMC Mol Biol. 2018 Dec 4;19:15. doi: 10.1186/s12867-018-0116-5 (PMC6278157; doi:10.1186/s12867-018-0116-5)
Supplement: Supplementary file 2 — Additional file 2. DNA manipulation steps in preparation for nested IPCR. [file 12867_2018_116_MOESM2_ESM.pdf]

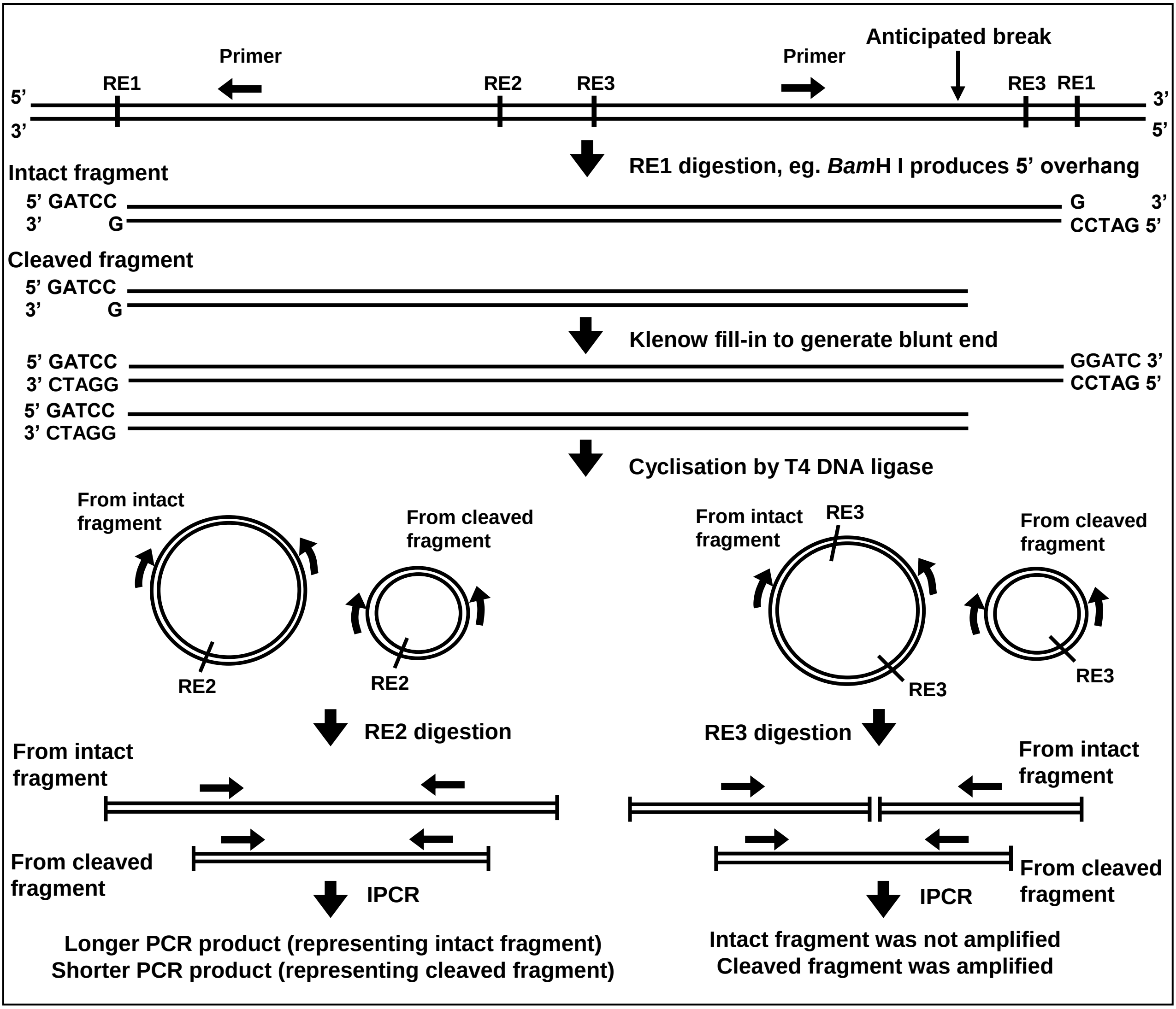

Additional file 2

DNA manipulation steps in preparation for nested IPCR. The extracted genomic DNA was modified through restriction enzyme digestion, Klenow fill-in and cyclisation. Nested IPCR was performed after these manipulation steps [26].
