# Supplementary material for: Matrix association region/scaffold attachment region (MAR/SAR) sequence: its vital role in mediating chromosome breakages in nasopharyngeal epithelial cells via oxidative stress-induced apoptosis
Source: BMC Mol Biol. 2018 Dec 4;19:15. doi: 10.1186/s12867-018-0116-5 (PMC6278157; doi:10.1186/s12867-018-0116-5)
Supplement: Supplementary file 3 — Additional file 3. DNA sequencing data. [file 12867_2018_116_MOESM3_ESM.pdf]

### $H_2O_2$-treated NP69 (breakpoint: 245566)

→ Direction of forward primer (AF9 245507 F)

```
         10        20        30        40        50
NATTGGTGTC AATCAAATGC TTTATGAACA TTTTAAAGTG ACACATTTTT    50
TAAAAGGTTA AGATCCGAAG TAAGAAAGAC GTATTTTCTG TCCTCATCTT   100
TATCTTTGGC TAGTGTGACT GCAGGGAAAA GTGGCTAATA CAAACATTTA   150
GATAACTTAC ACACAAAGCA AAAAGNNNNN N                       181
```

Direction of reverse primer (AF9 236211 R) ←

### $H_2O_2$-treated NP69 (breakpoint: 245591)

→ Direction of forward primer (AF9 245507 F)

```
         10        20        30        40        50
TNTATTGGTG TCNACAAATG CTTTATGAAC ATTTTAAAGT GACACATTTT    50
TTAAAAGGTT AAAGTTAATA AGGAAATGTA GTTAAATGAT CCGAAGTAAG   100
AAAGACGTAT TTTCTGTCCT CATCTTTATC TTTGGCTAGT GTGACTGCAG   150
GGAAAAGTGG CTAATACAAA CATTTAGATA ACTTACACAC AAAGCAAAAA   200
GTAGNANNGN NNT                                           213
```

Direction of reverse primer (AF9 236211 R) ←

### $H_2O_2$-treated NP69 (breakpoint: 245645)

→ Direction of forward primer (AF9 245507 F)

```
         10        20        30        40        50
TGTATTGGTG TCAATCAAAT GCTTTATGAA CATTTTAAAG TGACACATTT    50
TTTAAAAGGT TAAAGTTAAT AAGGAAATGT AGTTAAATGT CTTGTCAAAA   100
TAATCTCTTC AGATATATAT CTTTTAGTTA TTTCATTTCA TTGATCCGAA   150
GTAAGAAAGA CGTATTTTCT GTCCTCATCT TTATCTTTGG CTAGTGTGAC   200
TGCAGGGAAA AGTGGCTAAT ACAAACATTT AGATAACTTA CACACAAANN   250
NAAAAGNGNN NNN                                           263
```

Direction of reverse primer (AF9 236211 R) ←

## Additional file 3

**DNA sequencing data.** DNA sequencing was performed by using the forward and reverse primers that were used in the second round of IPCR of the *AF9* gene. The arrow above the sequence shows the direction of the forward primer (AF9 245507 F) while the arrow below the sequence shows the direction of the reverse primer (AF9 236211 R). The double arrow indicates the breakpoint. The nucleotide positions of the chromosomal breaks were mapped to the *AF9* sequence accessed from Ensembl database [EMBL:ENSG00000171843].

**$H_2O_2$-treated NP69 (breakpoint: 245659)**

→ Direction of forward primer (AF9 245507 F)

```
         10        20        30        40        50
TNTATTGGTG NNATCAAATG CTTTATGAAC ATTTTAAAGT GACACATTTT    50
TTAAAAGGTT AAAGTTAATA AGGAAATGTA GTTAAATGTC TTGTCAAAAT   100
AATCTCTTCA GATATATATC TTTTAGTTAT TTCATTTCAT TATCACCTTT   150
ATTTCGATCC GAAGTAAGAA AGACGTATTT TCTGTCCTCA TCTTTATCTT   200
TGGCTAGTGT GACTGCAGGG AAAAGTGGCT AATACAAACA TTTAGATAAC   250
TTACACACAA AGCAAAAAGT AGNNTNNNNN                         280
```

Direction of reverse primer (AF9 236211 R) ←

**$H_2O_2$-treated NP69 (breakpoint: 245711)**

→ Direction of forward primer (AF9 245507 F)

```
         10        20        30        40        50
CAAATGCTTT ATGAACATTT TAAAGTGACA CATTTTTTAA AAGGTTAAAG    50
TTAATAAGGA AATGTAGTTA AATGTCTTGT CAAAATAATC TCTTCAGATA   100
TATATCTTTT AGTTATTTCA TTTCATTATC ACCTTTATTT CCTGAATTCA   150
TTCTATTTAA TTTACTTTAT ACTTTTTTAT AATCCCAAAT ATAGATCCGA   200
AGTAAGAAAG ACGTATTTTC TGTCCTCATC TTTATCTTTG GCTAGTGTGA   250
CTGCAGGGAA AAGTGGCTAA TACNAACNTT TAGATAACTN NCNNACAAAG   300
CAAAANNNNN                                               310
```

←

**$H_2O_2$-treated NP69 (breakpoint: 245730)**

→ Direction of forward primer (AF9 245507 F)

```
         10        20        30        40        50
TATTGGTGTN CNTCAAATGC TTTATGAACA TTTTAAAGTG ACACATTTTT    50
TAAAAGGTTA AAGTTAATAA GGAAATGTAG TTAAATGTCT TGTCAAAATA   100
ATCTCTTCAG ATATATATCT TTTAGTTATT TCATTTCATT ATCACCTTTA   150
TTTCCTGAAT TCATTCTATT TAATTTACTT TATACTTTTT TATAATCCCA   200
AATATACTTT AATATTTATA ATCCAGATCC GAAGTAAGAA AGACGTATTT   250
TCTGTCCTCA TCTTTATCTT TGGCTAGTGT GACTGCAGGG AAAAGTGGCT   300
AATACAAACA TTTAGATAAC TTACACACAA AGCAAAAAGT AGAANNNNNN   350
N                                                        351
```

Direction of reverse primer (AF9 236211 R) ←

## $H_2O_2$-treated NP69 (breakpoint: 245804)

→ Direction of forward primer (AF9 245507 F)

```
         10        20        30        40        50
TATTGGTGTC AATCAAATGC TTTATGAACA TTTTAAAGTG ACACATTTTT    50
TAAAAGGTTA AAGTTAATAA GGAAATGTAG TTAAATGTCT TGTCAAAATA   100
ATCTCTTCAG ATATATATCT TTTAGTTATT TCATTTCATT ATCACCTTTA   150
TTTCCTGAAT TCATTCTATT TAATTTACTT TATACTTTTT TATAATCCCA   200
AATATACTTT AATATTTATA ATCCAGTACT GAGCCCTGTA GCCATAATGT   250
ACTTTTTATG ACTTTGTTCA ATTCAGTGTT CAGTTCAATG TTGCCGTTAG   300
ATCCGAAGTA AGAAAGACGT ATTTTCTGTC CTCATCTTTA TCTTTGGCTA   350
GTGTGACTGC AGGGAAAAGT GGCTAATACA AACATTTAGA TAACTTACAC   400
ACAAAGCAAA AAGTAGAANN                                    420
```

Direction of reverse primer (AF9 236211 R) ←

## $H_2O_2$-treated NP69 (breakpoint: 245817)

→ Direction of forward primer (AF9 245507 F)

```
         10        20        30        40        50
NGATTGGTGT NCATCAAATG CTTTATGAAC ATTTTAAAGT GACACATTTT    50
TTAAAAGGTT AAAGTTAATA AGGAAATGTA GTTAAATGTC TTGTCAAAAT   100
AATCTCTTCA GATATATATC TTTTAGTTAT TTCATTTCAT TATCACCTTT   150
ATTTCCTGAA TTCATTCTAT TTAATTTACT TTATACTTTT TTATAATCCC   200
AAATATACTT TAATATTTAT AATCCAGTAC TGAGCCCTGT AGCCATAATG   250
TACTTTTTAT GACTTTGTTC AATTCAGTGT TCAGTTCAAT GTTGCCGTTA   300
TAAATGTTGA GAAGATCCGA AGTAAGAAAG ACGTATTTTC TGTCCTCATC   350
TTTATCTTTG GCTAGTGTGA CTGCAGGGAA AAGTGGCTAA TACAAACATT   400
TAGATAACTT ACACACAAAG CAAAAAGTAG ANNNNNNNNN              440
```

Direction of reverse primer (AF9 236211 R) ←

## $H_2O_2$-treated NP69 (breakpoint: 245826)

→ Direction of forward primer (AF9 245507 F)

```
         10        20        30        40        50
CGGAGTGTGC GCTCTTATTG GTGTCAATCA AATTCTTGAT TGGTGTCCAT    50
CAAATGCTTT ATGAACATTT TAAAGTGACA CATTTTTTAA AAGGTTAAAG   100
TTAATAAGGA AATGTAGTTA AATGTCTTGT CAAAATAATC TCTTCAGATA   150
TATATCTTTT AGTTATTTCA TTTCATTATC ACCTTTATTT CCTGAATTCA   200
TTCTATTTAA TTTACTTTAT ACTTTTTTAT AATCCCAAAT ATACTTTAAT   250
ATTTATAATC CAGTACTGAG CCCTGTAGCC ATAATGTACT TTTTATGACT   300
TTGTTCAATT CAGTGTTCAG TTCAATGTTG CCGTTATAAA TGTTGAGAAG   350
ACAAAAGTGA TCCGAAGTAA GAAAGACGTA TTTTCTGTCC TCATCTTTAT   400
CTTTGGCTAG TGTGACTGCA GGGAAAAGTG GCTAATACAA ACATTTAGAT   450
AACTACACAC AAAGCAAAAA GAGCACGGAT                         480
```

Direction of reverse primer (AF9 236211 R) ←

## $H_2O_2$-treated NP69 (breakpoint: 245842)

→ Direction of forward primer (AF9 245507 F)

```
         10        20        30        40        50
NTATTGGTGT CAATCAAATG CTTTATGAAC ATTTTAAAGT GACACATTTT    50
TTAAAAGGTT AAAGTTAATA AGGAAATGTA GTTAAATGTC TTGTCAAAAT   100
AATCTCTTCA GATATATATC TTTTAGTTAT TTCATTTCAT TATCACCTTT   150
ATTTCCTGAA TTCATTCTAT TTAATTTACT TTATACTTTT TTATAATCCC   200
AAATATACTT TAATATTTAT AATCCAGTAC TGAGCCCTGT AGCCATAATG   250
TACTTTTTAT GACTTTGTTC AATTCAGTGT TCAGTTCAAT GTTGCCGTTA   300
TAAATGTTGA GAAGACAAAA GTGATTTTCC ACTATTTTGA TCCGAAGTAA   350
GAAAGACGTA TTTTCTGTCC TCATCTTTAT CTTTGGCTAG TGTGACTGCA   400
GGGAAAAGTG GCTAATACAA ACATTTAGAT AACTTACACA CAAAGNANNN   450
ANNNNCATNT NNGNNNNN                                      468
```

Direction of reverse primer (AF9 236211 R) ←

## $H_2O_2$-treated NP69 (breakpoint: 245959)

→ Direction of forward primer (AF9 245507 F)

```
         10        20        30        40        50
NNNNNNNNNN TNNNNTATTG GTGTCAATCA AATGCTTTAT GAACATTTTA    50
AAGTGACACA TTTTTTAAAA GGTTAAAGTT AATAAGGAAA TGTAGTTAAA   100
TGTCTTGTCA AAATAATCTC TTCAGATATA TATCTTTTAG TTATTTCATT   150
TCANNATCCC CTCNATTTCC TGAANTCNTT CTATTTAATT NACTTTATAC   200
TTTTNNATAA TCCCAAATAT NCTNTNATNT TTATANTCCA GTCCTGAGCC   250
CTGTAGCCAT AATGTACTTT TTAGGACTTT NTTCANTTCA GTCTCAGTTC   300
CATGNNGCCG TTATATATGN NGAGNAGACA AAAGTGATNT TCCCCNATNT   350
TAANTTTCCT ATGCTCTGCA CAGGAGGGTT NCTTCACACG TAACTGAANA   400
GTTCTGNCAA TANTCTCAAG NTCTCATCGN AACCTGACAN GNAGTGGTGT   450
NNCGATGNNC ACTGGTCAGA TCCGAAGTAA GAAAGACGTA TTTTCTGTCC   500
TCATCTTTAT CTTNGGNTAG TGTGACTGCA GGGAAAAGTG GCTAATACAA   550
ACATTTAGAT AACTACACAC AAGCAAAAGA G                       581
```

Direction of reverse primer (AF9 236211 R) ←

**$H_2O_2$-treated NP69 (breakpoint: 245970)**

Direction of forward primer (AF9 245507 F)

```
         10        20        30        40        50
NACAAATGCT TTATGAACAT TTTAAAGTGA CACATTTTTT AAAAGGTTAA    50
AGTTAATAAG GAAATGTAGT TAAATGTCTT GTCAAAATAA TCTCTTCAGA   100
TATATATCTT TTAGTTATTT CATTTCATTA TCACCTTTAT TTCCTGAATT   150
CATTCTATTT AATTTACTTT ATACTTTTTT ATAATCCCAA ATATACTTTA   200
ATATTTATAA TCCAGTACTG AGCCCTGTAG CCATAATGTA CTTTTTATGA   250
CTTTGTTCAA TTCAGTGTTC AGTTCAATGT TGCCGTTATA AATGTTGAGA   300
AGACAAAAGT GATTTTCCAC TATTTTAAAT TTCTTATGCT CTGCACAGGA   350
GGGTTACTTC ACAAGTAACT GAATAGTTCT GACAATAATC TCAAGATCTC   400
ATTGTAAACT GACAAGTAGT GGTGTTTCGA TGTTCATTGG TCAACTCCAA   450
TAAGGATCCG AAGTAAGAAA GACGTATTTT CTGTCCTCAT CTTTATCTTT   500
GGCTAGTGTG ACTGCAGGGA AAAGTGGCTA ATACAAACAT TTAGATAACT   550
TACACACAAA GCAAAANGNN NNNNN                              575
```

Direction of reverse primer (AF9 236211 R)

**$H_2O_2$-treated NP69 (breakpoint: 246089)**

Direction of forward primer (AF9 245507 F)

```
         10        20        30        40        50
NNTGGTGTNC AATCAAATGC TTTATGAACA TTTTAAAGTG ACACATTTTT    50
TAAAAGGTTA AAGTTAATAA GGAAATGTAG TTAAATGTCT TGTCAAAATA   100
ATCTCTTCAG ATATATATCT TTTAGTTATT TCATTTCATT ATCACCTTTA   150
TTTCCTGAAT TCATTCTATT TAATTTACTT TATACTTTTT TATAATCCCA   200
AATATACTTT AATATTTATA ATCCAGTACT GAGCCCTGTA GCCATAATGT   250
ACTTTTTATG ACTTTGTTCA ATTCAGTGTT CAGTTCAATG TTGCCGTTAT   300
AAATGTTGAG AAGACAAAAG TGATTTTCCA CTATTTTAAA TTTCTTATGC   350
TCTGCACAGG AGGGTTACTT CACAAGTAAC TGAATAGTTC TGACAATAAT   400
CTCAAGATCT CATTGTAAAC TGACAAGTAG TGGTGTTTCG ATGTTCATTG   450
GTCAACTCCA ATAACACTTA ATTATGGGCC AGTTCTATCA GGAAACTTTT   500
TTTCCTTCCT GTTTCTCTTC TGTGAGGAAT TACAGCTCTG AGCCCTCTCA   550
CTTGTAACAT TTTTCTTTCT CTACCCGTCA CTCTGATCCG AAGTAAGAAA   600
GACGTATTTT CTGTCCTCAT CTTTATCTTT GGCTAGTGTG ACTGCAGGGA   650
AAAGTGGCTA ATACAAACAT TTAGATAACT TACACACAAA GCAANNANNG   700
ANNNNNGNTN NNNNNN                                        716
```

Direction of reverse primer (AF9 236211 R)

**$H_2O_2$-treated HK1 (breakpoint: 245560)**

→ Direction of forward primer (AF9 245507 F)

```
         10        20        30        40        50
ATTGGTGTCA ATCAAATGCT TTATGAACAT TTTAAAGTGA CACATTTTTT    50
AAAAGATCCG AAGTAAGAAA GACGTATTTT CTGTCCTCAT CTTTATCTTT   100
GGCTAGTGTG ACTGCAGGGA AAAGTGGCTA ATACAAACAT TTAGATAACT   150
TACACGCAAG CGGG                                          164
```

Direction of reverse primer (AF9 236211 R) ←

**$H_2O_2$-treated HK1 (breakpoint: 245634)**

→ Direction of forward primer (AF9 245507 F)

```
         10        20        30        40        50
TGTATTGGTG TCAATCAAAT GCTTTATGAA CATTTTAAAG TGATACATTT    50
TTCAAAAGGT TAAAGTTAAT AAGGAAATGT AGTTAAATGT CTTGTCAAAA   100
TAATCTCTTC AGATATATAT CTTTTAGTTA TGATCCGAAG TAAGAAAGAC   150
GTATTTTCTG TCCTCATCTT TATCTTTGGC TAGTGTGACT GCAGGGAAAA   200
GTGGCTAATA CAAACATTTA GATAACTACA CACAAAGCAA AAAGTGACTA   250
```

Direction of reverse primer (AF9 236211 R) ←

**$H_2O_2$-treated HK1 (breakpoint: 245681)**

→ Direction of forward primer (AF9 245507 F)

```
         10        20        30        40        50
ATCTTATTGG TGTCCATCAA ATGCTTTATG AACATTTTAA AGTGACACAT    50
TTTTTAAAAG GTTAAGTTAA AAAGGAATCG AGTAAAGATT TATGAACATA   100
TTAAAGTTCA CATTTATACA TAGGTTAAAG GCATAAGGAA ATGTAGAAAA   150
ATATCTTGTC ATAATATTTT TATCAGATAT TCATAAATTC ATTTTTTCGT   200
TATATCATTT CCTTGACTCC TTGATTTCAT TTTTTCTGTT TACTTAATTT   250
GATCCGAAGT AAGAAAGACG TATTTTCTGT CCTCATCTTT ATCTTTGGCT   300
AGTGTGACTG CAGGGAAAAG TGGCTAATAC AAACATTTAG ATAACTACAC   350
ACAAAGCAAA AAGAGAATGT TCG                                373
```

Direction of reverse primer (AF9 236211 R) ←

**$H_2O_2$-treated HK1 (breakpoint: 245755)**

→ Direction of forward primer (AF9 245507 F)

```
         10        20        30        40        50
TTCCTCAAAT GCTTTATGAA CATTTTAAAG TGACACATTT TTTAAAAGGT     50
TAAAGTTAAT AAGGAAATGT AGTTAAATGT CTTGTCAAAA TAATCTCTTC    100
AGATATATAT CTTTTAGTTA TTTCATTTCA TTATCACCTT TATTTCCTGA    150
ATTCATTCTA TTTAATTTAC TTTATACTTT TTTATAATCC CAAATATACT    200
TTAATATTTA TAATCCAGTA CTGAGCCCTG TAGCCATAAT GTGATCCGAA    250
GTAAGAAAGA CGTATTTTCT GTCCTCATCT TTATCTTTGG CTAGTGTGAC    300
TGCAGGGAAA AGTGGCTAAT ACAAACATTT AGATAACTTC ACACAGGCGG    350
TAGAGANAG                                                 359
```

Direction of reverse primer (AF9 236211 R) ←

**$H_2O_2$-treated HK1 (breakpoint: 245949)**

→ Direction of forward primer (AF9 245507 F)

```
         10        20        30        40        50
TATTGGTGTC AATCAAATGC TTTATGAACA TTTTAAAGTG ACACATTTTT     50
TAAAAGGTTA AAGTTAATAA GGAAATGTAG TTAAATGTCT TGTCAAAATA    100
ATCTCTTCAG ATATATATCT TTTAGTTATT TCATTTCATT ATCACCTTTA    150
TTTCCTGAAT TCATTCTATT TAATTTACTT TATACTTTTT TATAATCCCA    200
AATATACTTT AATATTTATA ATCCAGTACT GAGCCCTGTA GCCATAATGT    250
ACTTTTTATG ACTTTGTTCA ATTCAGTGTT CAGTTCAATG TTGCCGTTAT    300
AAATGTTGAG AAGACAAAAG TGATTTTCCA CTATTTTAAA TTTCTTATGC    350
TCTGCACAGG AGGGTTACTT CACAAGTAAC TGAATAGTTC TGACAATAAT    400
CTCAAGATCT CATTGTAAAC TGACAAGTAG TGGTGTTTCG ATGTGATCCG    450
AAGTAAGAAA GACGTATTTT CTGTCCTCAT CTTTATCTTT GGCTAGTGTG    500
ACTGCAGGGA AAAGTGGCTA ATACAAACAT TTAGATAACT TACACACAAA    550
GCAAAAAGTA GAA                                            563
```

Direction of reverse primer (AF9 236211 R) ←
